# The *Drosophila* ecdysone receptor promotes or suppresses proliferation according to ligand level

**DOI:** 10.1016/j.devcel.2023.08.032

**Published:** 2023-09-27

**Authors:** Gantas Perez-Mockus, Luca Cocconi, Cyrille Alexandre, Birgit Aerne, Guillaume Salbreux, Jean-Paul Vincent

**Affiliations:** 1 The Francis Crick Institute, London NW1 1AT, UK; 2 Department of Genetics and Evolution, University of Geneva, Quai Ernest-Ansermet 30, 1205 Geneva, Switzerland

## Abstract

The steroid hormone 20-hydroxy-ecdysone (20E) promotes proliferation in *Drosophila* wing precursors at low titer but triggers proliferation arrest at high doses. Remarkably, wing precursors proliferate normally in the complete absence of the 20E receptor, suggesting that low-level 20E promotes proliferation by overriding the default anti-proliferative activity of the receptor. By contrast, 20E needs its receptor to arrest proliferation. Dose-response RNA sequencing (RNA-seq) analysis of *ex vivo* cultured wing precursors identifies genes that are quantitatively activated by 20E across the physiological range, likely comprising positive modulators of proliferation and other genes that are only activated at high doses. We suggest that some of these “high-threshold” genes dominantly suppress the activity of the pro-proliferation genes. We then show mathematically and with synthetic reporters that combinations of basic regulatory elements can recapitulate the behavior of both types of target genes. Thus, a relatively simple genetic circuit can account for the bimodal activity of this hormone.

## Graphical abstract

**Graphical abstract:**
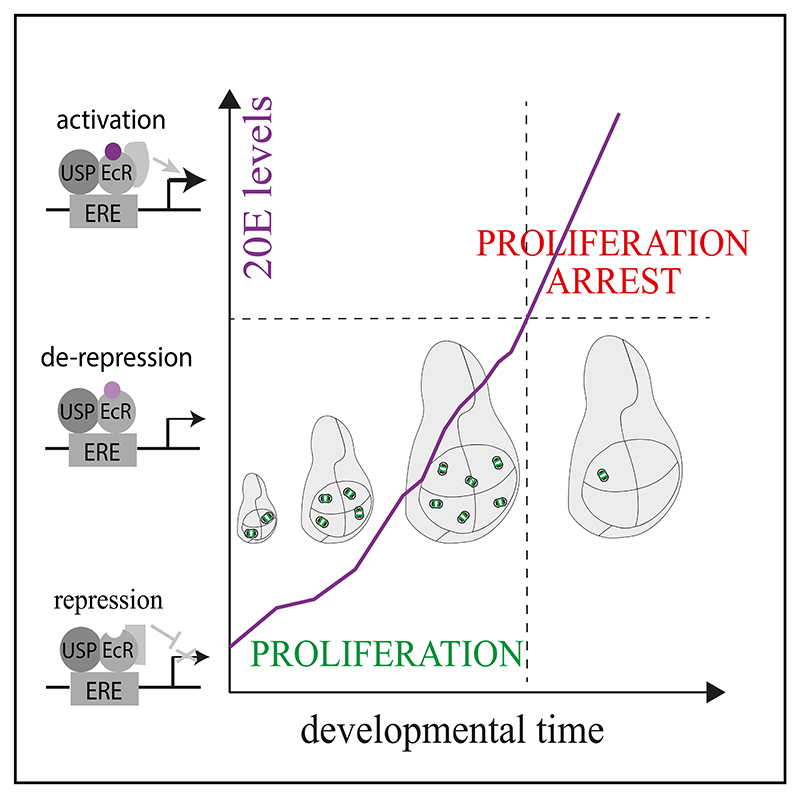

## Introduction

Type II nuclear receptors constitute a subclass of nuclear receptors, transcription factors that bind small lipophilic molecules and mediate their signaling activity during development and adult homeostasis. One example is the retinoic acid receptor, which regulates germ layer formation, body axis formation, neurogenesis, cardiogenesis, and many other processes during vertebrate development.^1–3^ Although type I receptors are regulated by ligand-induced nuclear import, type II receptors are permanent residents of the nucleus. They bind DNA as heterodimers (e.g., with the retinoid X receptor),^4^ which, in the absence ligand, recruit a corepressor. Upon ligand binding, the corepressor is replaced by a coactivator to trigger transcription. Thus, type II nuclear receptors can act either as transcription repressor or activator. It is generally thought that they regulate a wide range of activities by interacting with tissue- or stage-specific co-factors.^5^ In some case, type II receptors seem to have opposite effects on the same process, e.g., stimulating or suppressing proliferation, depending on the context.^6–8^ The molecular basis of this feature remains poorly understood.

**Figure 1:**
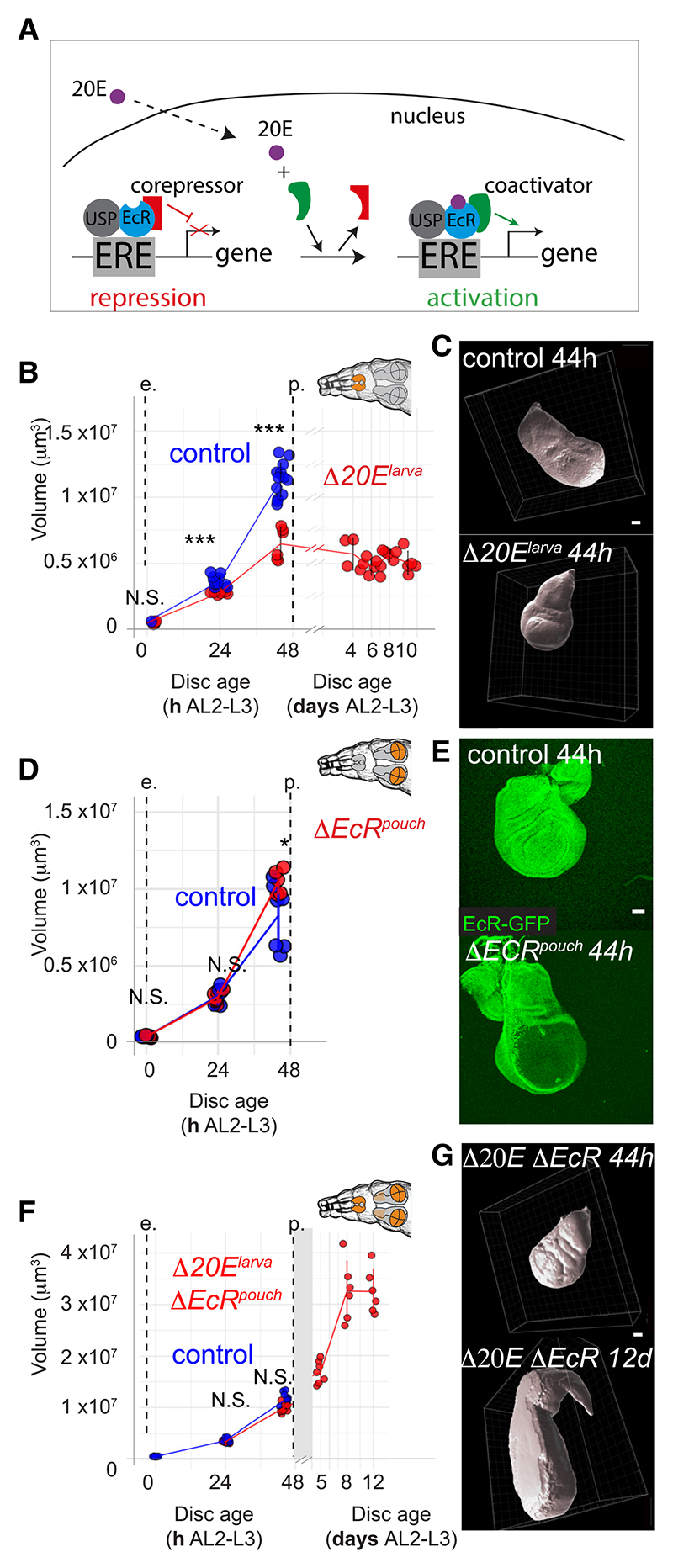
20E and EcR have opposite effects on wing disc growth (A) Schematic representation of EcR activity as a function of ligand availability. EcR recruits a transcriptional corepressor in the absence of 20E (left) or a coactivator in the presence of 20E (right). (B and C) Inhibition of 20E synthesis (Δ*20E^larva^*: *phtm>Oct*β*3R^i^*), impairs wing disc growth (quantification of volumetric reconstruction and representative images are shown, as in (D)–(G) below. n ≥ 5 discs for each time point, except for the data at 4 days AL2-L3, where n = 4). (D and E) Pouch-specific inactivation of EcR (Δ*EcR*^pouch^: *EcR*^KO^/*EcR*^Cko^ *pdm2-Gal4 UAS-Flp*) has no significant impact on disc growth (n ≥ 5). (F and G) Pouch-specific inactivation of EcR allows growth even when 20E synthesis is inhibited (Δ*20E^larva^* Δ*EcR*^pouch^:*EcR*^KO^ *LexOP-Flp/EcR-GFP^cKO^; rotund-LexA UAS-Octb3R^RNAi^/phtm-Gal4*;). Note that the notum also pro-liferates in this background, perhaps as an indirect consequence of EcR inactivation in adult muscle precursors, where *rotund* is expressed.^14^ In this background, pupariation does not take place, allowing sustained disc growth beyond the normal time of pupariation (n ≥ 4 discs). All error bars represent standard deviation. * p < 0.5, *** p < 0.01, N.S., no statistical difference. Wil-coxon Ranks sum tests were performed in (B), (D), and (F). Scale bars represent 50 μm.

The main type II nuclear receptor of *Drosophila* is the ecdysone receptor (EcR). EcR binds to DNA as a heterodimer with Ul-traspiracle (USP) and, in the absence of ligand, recruits transcriptional corepressors such as Smrter^9–13^ (Figure 1A). Ligand binding induces a conformational change that allows the recruitment of transcriptional coactivators.^10,15–18^ Production of the active hormone starts in the prothoracic gland (PG) through the concerted activity of enzymes encoded by the so-called halloween genes, which are themselves regulated by a combination of developmental, nutritional, and stress signals.^19–23^ These enzymes transform cholesterol into ecdysone (E), which is secreted from the PG to reach the larval fat body and gut, where the hydroxyl-transferase encoded by *shade*^24^ converts it into the active form, 20-hydroxy-ecdysone (20E). 20E is then released into the circulation and gains access to target tissues through a dedicated transporter.^25^

Depletion of systemic 20E by genetic manipulation of the PG leads to a marked slowdown of proliferation in wing precursors,^26–31^ suggesting that 20E is a proliferative signal. By contrast, at the onset of metamorphosis, 20E leads to G2 arrest, in preparation for the morphogenetic rearrangements that sub-sequently take place.^32,33^ Therefore, 20E seems to promote pro-liferation during larval stages and prevent proliferation at pupariation. How can the same molecular signal drive opposite effects on proliferation? We first confirm with a conditional allele the earlier suggestion that 20E promotes growth by preventing EcR from inhibiting growth/proliferation.^34,35^ We also show with a calibrated reporter of EcR activity that low-level 20E stimulates proliferation *ex vivo*, whereas high levels are inhibitory. RNA sequencing (RNA-seq) analysis suggests that proliferation is actively suppressed by one or several “high-threshold” genes, which dominantly suppress the pro-proliferation genes that respond to the whole range of physiological 20E concentrations. Mathematical modeling, validated by synthetic reporters, shows that relatively simple changes in the *cis*-regulatory region of target genes could account for the qualitatively distinct responses of 20E target genes.

## Results

### Ecdysone is not required for tissue growth if EcR is genetically ablated

As a prelude to assessing the role of 20E and its receptor in growth and proliferation, we measured the growth of wild-type *Drosophila* wing imaginal discs during the 3^rd^ instar, using volume as a proxy for biomass.^36^ To accurately stage imaginal discs, larvae were selected at the L2-L3 transition, a well-defined developmental milestone, then allowed to grow for specific periods of time before the discs were dissected, fixed, stained with DAPI, and mounted in a drop of agar containing a clearing agent. The volume was then calculated from three-dimensional (3D) reconstructed confocal stacks^37^ (Figures S1A and S1B). This analysis revealed that, during the 48 h of the 3^rd^ instar, wild-type wing imaginal disc volume increases by about 27-fold, which agrees with previous reports suggesting that, during this time, imaginal discs cells undergo approximately 5–6 divisions during the 3^rd^ instar.^38–42^ We cannot be sure that growth terminates at pupariation since extensive morphogenesis occurring at this time makes volume measurements difficult. However, it is clear that, at the end of the 3^rd^ instar, only occasional cells undergo mitosis, as assayed with anti-pH3 staining^33,43^, can be detected, suggesting that proliferation grinds to a halt at the onset of pupariation.

Using the above assay, we reassessed the effect of reducing systemic 20E levels on wing imaginal disc growth and proliferation during the 3^rd^ instar. The precursor of 20E, E, is produced by the PG, which integrates inputs from various signals, including that mediated by the β3-octopamine receptor.^44^ E20, the active hormone is then produced by oxidization in peripheral tissue. We confirmed previous findings^44^ that larvae expressing an RNAi transgene against the β3-octopamine receptor specifically in the PG fail to metamorphose (Figure S1C), an indication that 20E controls this developmental transition. The wing imaginal discs within these animals grew poorly, especially during the second half of the 3^rd^ instar. They also failed to gain volume during the subsequent 8 days of extended larval period (Figures 1B and 1C). Note, however, that this genetic manipulation does not completely abrogate 20E production since these animals do progress through earlier instars, most likely as a result of sustained 20E during this early period.^44^ In this genetic background (*phtm-Gal4 UAS-Octb3R^RNAi^*), 20E is markedly reduced from the mid 3^rd^ stage (Ohhara et al.^44^; see also evidence from a signaling reporter below), and this correlates with strong growth reduction at this time. These observations confirm and extend the various reports that 20E is essential for imaginal discs growth.^27–31,45,46^

The requirement of 20E for imaginal discs growth seems at odds with other reports that RNAi-mediated knockdown of the 20E receptor specifically in imaginal discs does not impair growth.^34,35,47^ Since RNAi may leave residual gene activity, we engineered the *EcR* locus so that it can be completely inactivated by Flp recombinase in a tissue-specific manner (Figure S1D). A DNA fragment encoding GFP was inserted at the 3′ end of the coding region, and FRT (Flp recognition target) sites were added to allow Flp-mediated excision of the last four exons and the additional GFP-coding sequences. The product of this allele, termed EcR-GFP^cKO^ was found in the nucleus (Figure S1F), where the endogenous EcR is known to reside.^48^ Moreover, EcR-GFP^cKO^ homozygous flies showed no morphological defects and developed at the same rate as control, wild-type larvae (Figure S1E). We conclude therefore that, in the absence of Flp, this allele is fully functional. We then used *pdm2-Gal4* with *UAS-Flp* to inactivate this allele in the prospective wing of hemizygous *EcR* larvae (*EcR-GFP^cKO^/EcR^KO^; pdm2-Gal4 UAS-Flp*). This had no effect, either on developmental timing of the whole larva (Figure S1G) or on the rate of imaginal disc growth, and 3^rd^ instar imaginal discs lacking EcR grew at the same rate as wild-type disc (Figures 1D and 1E). We therefore conclude that EcR is not required for imaginal disc growth, even though its ligand is.

One could explain the requirement of 20E, but not that of EcR, for imaginal disc growth by invoking the existence of a distinct receptor through which 20E would control growth. To assess this possibility, we created larvae that are impaired in E (and hence 20E) production (*phtm-Gal4 UAS-Octβ3R^RNAi^*) while at the same time lacking, from the time of the L2-L3 transition, EcR in the wing pouch (*rotund-LexA LexOP-Flp EcR-GFP^cKO^/EcR^KO^*), named hereafter Δ*20E^larva^* Δ*EcR^pouch^*. In contrast to the situation with Octβ3R downregulation alone (Δ*20E^larva^*), wing imaginal discs from Δ*20E^larva^* Δ*EcR*^pouch^ animals grew seemingly normally during the usual growth period, showing that 20E is not absolutely required for growth and hence that an alternative receptor is not involved. As expected from the loss of 20E, Δ*20E^larva^* Δ*EcR*^pouch^ larvae failed to pupariate, providing extra time for growth. This allowed imaginal discs to reach 3 times the size of wild-type discs 10 days after the onset of the 3^rd^ instar (Figures 1F and 1G). Thus, EcR is required for proliferation arrest, most likely in response to the pulse of 20E at pupariation. Accordingly, imaginal discs expressing an EcR^RNAi^ transgene at the time of disc specification (with *vg-Gal4 UAS-Flp Act5c-FRT-STOP-FRT-Gal4*) within larvae producing 20E normally overgrew somewhat beyond the time of pupariation (Figures S1H–S1J). These observations suggest that, in the absence of 20E, EcR could act as a proliferation brake. During the growth period, 20E would release this brake, thus promoting proliferation. By contrast, at the end of the 3^rd^ instar, 20E would activate EcR to trigger the morphogenetic anti-proliferation program of pupariation.^32,49,50^ How could the same ligand have opposite effects on proliferation? One possibility is that low levels of 20E promote growth and proliferation during the 3^rd^ instar, whereas the high levels present at pupariation trigger proliferation arrest and morphogenesis.

### EcR activity rises during the 3^rd^ instar

**Figure 2:**
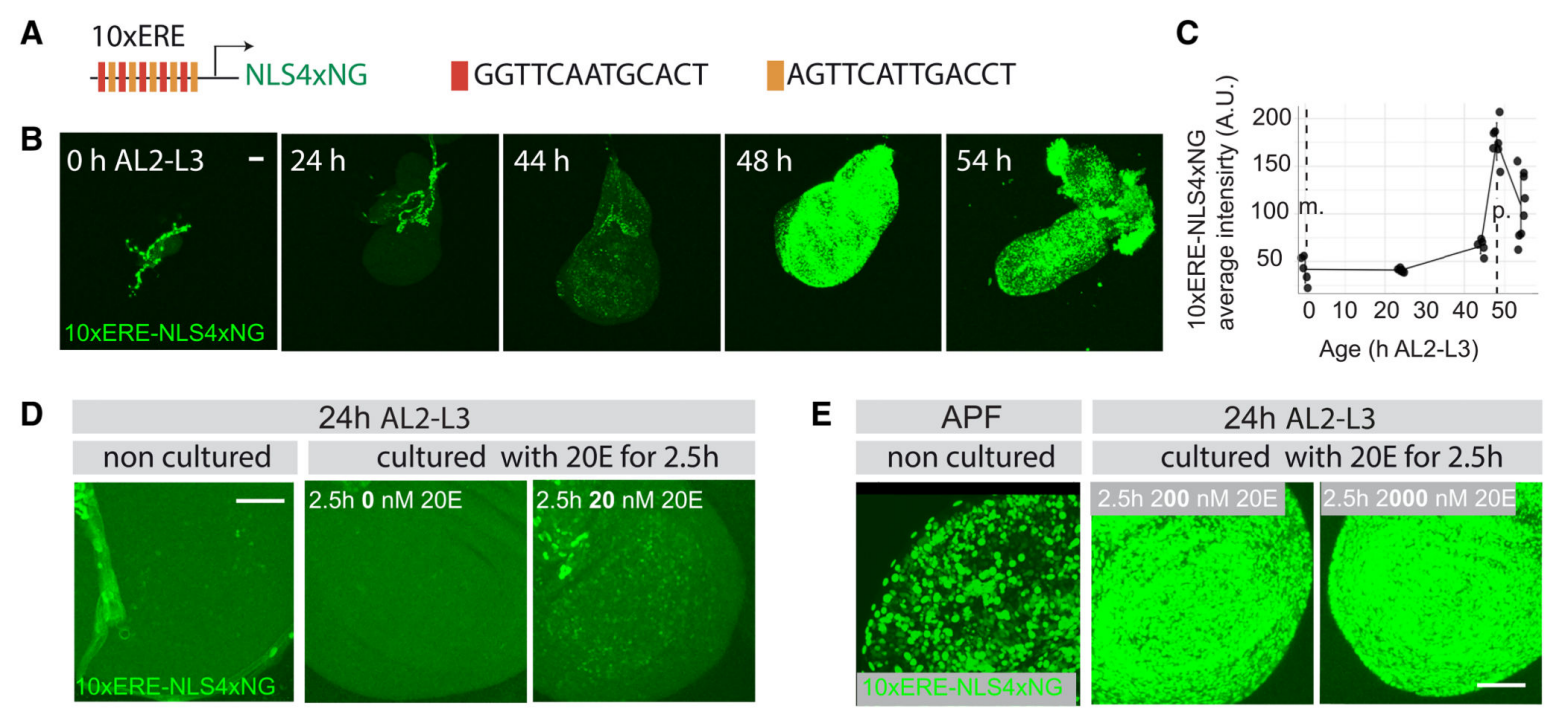
Effective 20E levels rise during the 3^rd^ instar (A) Schematic representation of the 10xERE-NLS4xNG reporter. (B and C) Representative micrographs (all taken under identical conditions) and quantification of reporter fluorescence (n ≥ 5 wing discs for each of the time points). Non-specific fluorescence in the trachea, seen in all the NLS4xNG reporters made so far has been excluded from quantification. (D and E) Estimation of *in vivo* 20E level at 24 h AL2-L3 (mid L3) and at the onset of pupariation. Reporter fluorescence intensity in discs explanted at 24 h AL2-L3 and cultured for 2.5 h with 20, 200, and 2,000 nM 20E was compared with that in discs freshly explanted at 24 h AL2-L3 or at pupariation. Each micrograph is representative of ≥5 acquired. Error bars represent standard deviation, and scale bars represent 50 μm. m. stands for molting and p. for pupariation.

Liquid chromatography-mass spectrometry (LC-MS) measurements suggest that imaginal discs are exposed to relatively low 20E levels during the 3^rd^ instar compared with pupariation.^51^ To estimate to what extent systemic levels of 20E translate in EcR signaling activity within imaginal discs, we devised a reporter comprising five alternating copies of two consensus EcR response element (ERE)^48,52^ upstream of a minimal heat shock promoter driving the transcription of a DNA fragment encoding a nuclear-targeted NeonGreen tetramer (10xERE-NLS4xNG) (Figure 2A). Nuclear fluorescence was readily detected in transgenic imaginal discs at the end of the 3^rd^ instar. This signal was abrogated by the expression of a dominant negative form of EcR (Figure S2A). Fluorescence was also detected in live pupae in a temporal pattern that mimics the 20E dynamics previously determined by LC-MS (compare Figure S2B and Video S1 with data from Lavrynenko et al.^51^). These data show that 10xERE-NLS4xNG is a reporter of EcR activity. Note, however, the absence of NeonGreen fluorescence at the very onset of the 3^rd^ instar (Figures 2B and 2C; ignore fluorescence in the trachea, which is due to non-specific activity of NLS4 xNG reporters), when low-level 20E are present, suggesting that 10xERE-NLS4xNG has limited sensitivity (a more sensitive reporter based on EcR de-repression is described below). The pattern of fluorescence from 10xERE-NLS4xNG confirms the expectation that imaginal discs are exposed to an increasing level of 20E during the 3^rd^ instar and that proliferation arrest correlates with particularly high signaling activity. Therefore, as previously suggested for lepidopterans,^53–55^ different levels of 20E could trigger distinct effects on proliferation (promotion at a low level, inhibition at a high level).

### 20E level determines whether EcR promotes or suppresses proliferation *ex vivo*

To estimate the 20E dose response on growth and proliferation, we turned to *ex vivo* explants, which can be exposed to known concentrations of 20E. First, we calibrated the effective concentration of 20E that discs are exposed to *in vivo* by measuring the effect of 20E on 10xERE-NLS4xNG activity in explanted mid 3^rd^ instar discs. Immediately after dissection (non-cultured in Figure 2D), weak but detectable fluorescence was present. In the absence of added 20E, this signal decayed to background level within 2.5 h of culture (Figure 2D, middle), indicating that reporter activity is not sustained in the absence of 20E. By contrast, addition of 20 nM 20E led to an increase in reporter activity (Figures 2D, right, and 2E), which peaked at 2.5 h before dropping down (Figures 2C and 2D). These observations suggest that, at the time of dissection (24 h after L2 to L3 transition or AL2-L3), *in vivo* concentration of 20E is lower than 20 nM. The delay before peak expression could be caused by the time needed for transcription, translation, and folding of Neon Green, whereas subsequent decay of the signal could reflect suboptimal culture conditions and/or 20E depletion over time. Based on these results, we opted to measure the reporter’s dose response after 2.5 h in culture. Culture with 200 or 2,000 nM of 20E (Figures 2E, middle and right, and S2F) led to the same, strong signal, suggesting saturation of reporter activity above 200 nM. Similarly strong reporter activity was seen in freshly explanted pupariating discs (Figure 2E, left), suggesting that, at this stage, *in vivo* 20E concentration is 200 nM or higher. We conclude therefore that, *in vivo*, imaginal discs experience 20E at a concentration below 20 nM at the onset of the growth period and around 200–2,000 nM at the time of pupariation. This agrees broadly with LC-MS measurements, which suggest that peak 20E concentration at pupariation is about 140 times higher than at the mid 3^rd^ instar.^51^

**Figure 3:**
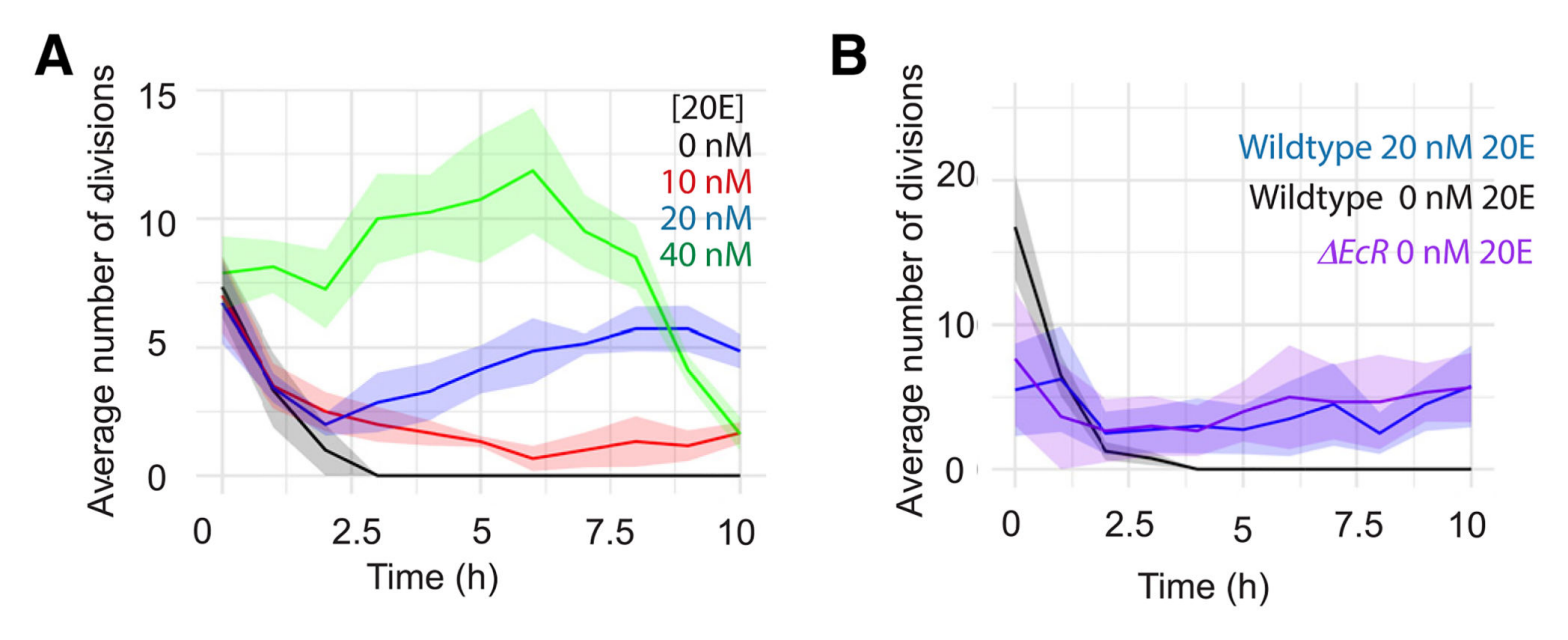
20E concentrations ranging from 10 to 40 nM promote proliferation *ex vivo* (A) Number of divisions measured in a region of interest (ROI) of wing disc explants cultured with the indicated 20E concentration. In the absence of 20E, the discs stop proliferation (n = 3) within 2.5 h of culture. Adding 10 (n = 6), 20 (n = 7), or 40 nM (n = 8) 20E rescues proliferation in a concentration-dependent manner. (B) Inactivation of EcR (n = 3) allows explanted discs to proliferate at the same rate as wild-type discs exposed to 20 nM 20E (n = 4), significantly faster than discs cultured in 0 nM 20E (n = 4). The average number of mitoses was calculated using a rolling 1 h period. Error bars represent standard error to the mean.

Having established the range of 20E concentrations that imaginal discs are exposed to, we proceeded to assess how 20E affects proliferation in explanted mid 3^rd^ instar imaginal discs. As mentioned above, in the absence of 20E, proliferation ceased within 2.5 h in culture (Figure 3A; Video S2A). This could be rescued, in a dose-dependent manner, by addition of 10, 20, or 40 nM 20E in the culture medium (Figure 3A; Videos S2B–S2D). Proliferation was also sustained in EcR-null imaginal discs without added 20E (Figure 3B; Video S3), in accordance with the finding that EcR is dispensable for growth *in vivo*. The rate of proliferation in explanted EcR mutant discs was similar to that in explanted wild-type discs treated with 20 nM 20E (Figure 3B), suggesting that sub-20 nM 20E suffices to overcome the repressive influence of EcR on proliferation, whereas higher concentrations (20 and 40 nM) could provide a further boost. No such boost was seen in EcR mutant discs treated with 40 nM 20E (not shown). These results clearly demonstrate that 20E promotes imaginal disc proliferation *ex vivo*, in accordance with previous reports,^26,29^ and the effect of 20E depletion β3-octopamine receptor knockdown *in vivo* (see above). However, upon exposure to 2,000 nM 20E, mitotic figures were no longer detected and early signs of eversion could be seen, despite the early stage (mid 3^rd^ instar) (Video S2E). This effect of high concentration 20E is dependent on EcR since no sign of eversion could be seen in mid 3^rd^ instar *EcR* mutant discs treated with 2,000 nM 20E (Figure S3). Therefore, 20E has a bimodal effect, promoting proliferation at low concentration while preventing proliferation at high concentration. Thus, 20E could trigger qualitatively distinct transcriptional responses at different concentrations.

### Dose-dependent effects of 20E on transcriptional activity

**Figure 4:**
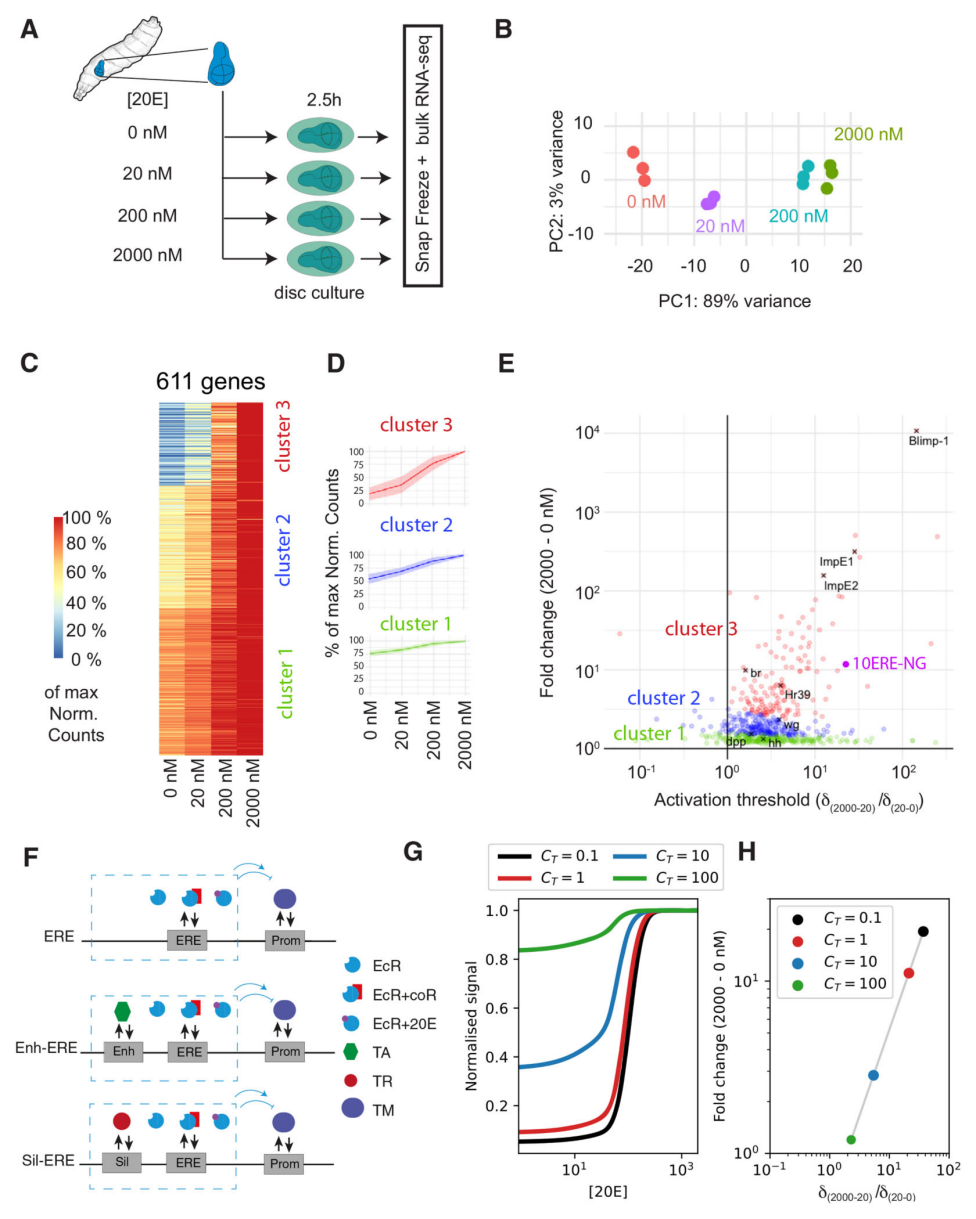
20E target genes have various thresholds of activation (A) Experimental protocol to assess the transcriptional response to different 20E concentrations. (B) Principal component analysis of the RNA-seq results shows clustering of the biological replicates. (C) Transcriptional response of the 611 genes that are upregulated in response to increasing concentrations of 20E. Expression level is normalized to the highest value; normalization according to lookup table on the left. The gene responses were organized in three clusters as shown. (D) Average response of each cluster, with standard deviation represented by a lightly colored ribbon. (E) Map displaying the extent of up-regulation of the 611 genes across the concentration range. The abscissa shows up-regulation in the 20– 2,000 nM range relative to that in the 0–20 nM range (a high value reflects a gene that is mostly activated at high concentration). This is plotted in relation, in ordinate, to the overall fold change of expression between 0 and 2,000 nM. Genes are color-coded according to the cluster they belong to. Specific genes of interest are indicated with black crosses. (F) Diagrammatic representation of regulation of three hypothetical target genes considered by the thermodynamic model, which differ by the presence or absence of a constitutive enhancer (Enh) or silencer (Sil), to which transcriptional activators (TAs) or transcriptional repressors (TRs) can bind. In the model, EcRs bound to ERE act as activators when associated with 20E, recruiting the TM to the promoter. EcRs bound to ERE act as repressors when associated with their corepressor (coR), inhibiting TM recruitment and transcriptional activity. (G) Transcriptional activity as a function of ecdysone concentration predicted by the thermody-namic model, normalized to the maximum, for a gene regulated by an ERE (red curve), in the presence of a constitutive enhancer of increasing strength (blue and green curves), and in the presence of a constitutive silencer (black curve). The enhancer leads to increased normalized baseline activity at low 20E, as well as a decrease of the threshold ecdysone concentration at which the genes expression level changes. $\kappa_{P}=0.1,{\bar{\kappa }}_{R}=1,C_{ER}=0.1,C_{EA}=10$ See Methods S1 for parameter definitions. (H) Predicted relationship between fold change increase in transcriptional rate between 0 and 2,000 nM (y axis) and relative increase in gene expression at 2,000–20 nM vs. 20–0 nM ecdysone concentration (x axis), as the strength of the constitutive enhancer is varied. Colored dots correspond to curves in (G).

To assess the transcription response to different concentrations of 20E, mid 3^rd^ instar wing imaginal discs were cultured for 2.5 h in 0, 20, 200, or 2,000 nM 20E and then processed for mRNA-seq (Figure 4A) and data analysis. As shown in Figure 4B, a single principal component, which accounted for 89% of the variance,could reliably distinguish the four samples. As further evidence for the quality of the data, known targets of EcR signaling were found to be expressed in a concentration-dependent manner (Figure S4A). This was confirmed by immunofluorescence for Br-Z1-GFP^34,35^ and Blimp-1-GFP^56^ (Figures S4B–S4E). These observations give confidence that the transcriptional response of explanted mid 3^rd^ instar discs is physiologically relevant and warrants further analysis.

For a first level of analysis, we used DESEQ2 to identify genes whose changes in expression could be explained by dose dependence on 20E (see STAR Methods). Among the 1,489 resulting genes (Table S1), we focused our attention on those changing monotonically and with a total read number exceeding a small arbitrary threshold (see STAR Methods for further details). This first selection identified 611 genes that are upregulated in a 20E concentration-dependent manner and 635 genes that are down-regulated. Since 20E-bound EcR acts as a transcriptional activator,^10^ we surmised that the downregulated genes are repressed indirectly.^52^ Indeed, these genes had relatively few EcR binding sites in the vicinity of their transcription start site (Figures S4F and S4G). By contrast, the set of upregulated genes is characterized by an enrichment of canonical EcR binding sites (Figures S4F and S4G). We therefore chose to restrict subsequent analysis to the 611 upregulated genes, which are most likely controlled directly by EcR and 20E.

For each gene, reads were normalized to the highest value at any of the four concentrations, thus allowing the different dose responses to be compared despite wide ranges in expression levels. The results were then plotted on a heatmap (Figure 4C). K-medoids clustering of normalized gene expression revealed that 20E target genes could be classified in three clusters according to the shape of their response (Figure 4D). Although such classification is somewhat arbitrary, as the clusters follow a continuous spectrum, it highlights qualitative differences in the dose response of 20E target genes. Thus, cluster 1 genes are expressed in a dose-dependent manner at all 20E levels, but with significant basal expression in the absence of 20E. Cluster 3 genes (red), by contrast, are not expressed in the absence of 20E and respond with a high dynamic range across the spectrum of concentrations. These will be referred to as high-threshold genes by analogy to the genes that respond only to high-level morphogen signaling. Finally, cluster 2 (blue) showed an intermediate response. The behavior of representative genes from each of the three clusters is shown in Figure S4H. To determine, for any given gene, whether the dynamic range of the response lies mostly at high or low 20E concentrations, we devised the δ_(2,000–20)_/δ_(20–0)_ ratio, which compares the change in gene expression between 20 and 2,000 nM to that between 0 and 20 nM. This parameter was plotted against the overall fold change (Figure 4E), defined as the ratio of expression levels at 2,000 and 0 nM. In the resulting response map, high-threshold cluster 3 genes (red dots) appear mostly on the upper-right side. By contrast, cluster 1 genes (green) tend to display a relatively low overall fold change and therefore appear at the bottom of the map. This map shows a weak but significant correlation (R = 0.28) between the overall fold change and the δ_(2,000-20)_/δ_(20-0)_ ratio. By comparison, no such correlation could be seen with a dataset of randomly generated virtual genes (R = −0.043) (Figures S4I and S4J), suggesting that the correlation between overall fold change in gene expression and the tendency to respond mostly to high 20E concentrations, as seen in the feature map, is genuine. In conclusion, RNA-seq analysis reveals a range of transcriptional responses, which could underlie the bimodal effect of 20E on proliferation. Thus, we expect genes involved in termination of proliferation to fall in the upper-right side of the response map (mostly expressed at high 20E concentrations). Pro-proliferation genes could possibly be found in the rest of the map, i.e., in the lower-left side (active at low 20E concentrations), although we cannot exclude the possibility that targets not considered by our analysis could mediate the pro-proliferation activity of 20E (see discussion).

### Emulating EcR target gene behaviors *in silico* and *in vivo*

The 10xERE-NLS4xNG reporter, which receives its inputs only from EcR and 20E, is unlikely to recapitulate the range of dose responses of EcR target genes. Calculation of the δ_(2,000-20)_/ δ_(20-0)_ ratio places this minimal reporter within cluster 3 (high-threshold targets, Figure 4E, purple dot). What are the regulatory features that would allow a reporter gene to mimic the range of behavior seen with natural EcR-responsive genes? Cluster 1 genes are characterized by non-zero baseline activity. They must therefore receive a positive input from a separate enhancer element. At the other extreme, high-threshold genes remain largely silent in the 0–20 nM range of 20E concentrations. This could be achieved by a silencer element that only allows expression at high concentrations of 20E.

To mathematically explore how such simple elements would alter the response of 10xERE-NLS4xNG, we devised a coarse-grained thermodynamic model of transcriptional regulation that combines the effect of EREs to that of constitutive enhancer/ silencer elements (see Methods S1). In this model, an average transcriptional activity is derived from the probability of recruitment of the transcriptional machinery (TM) to the promoter of the gene.^57–61^ The affinity of the TM for the promoter depends on the presence of the constitutive activator or repressor, as well as whether EcR is associated with its coactivator or corepressor. Competitive binding of the coactivator or corepressor to EcR was incorporated in a simple model that tracks the probability of the possible complexes (see schematic of Figures 4F, S5A, and S5B).^9–13,15–18^ This model successfully recapitulates the previously reported “sponge effect” of an EcR fragment lacking its DNA binding domain^62^ (Figure S5C). We further assume for simplicity that all EREs are occupied by EcR, that the corepressor has a fixed concentration, and that the coactivator is not required for activation of EcR. We find first order Hill-type functional forms for *P*_act_(*E*) and *P*_rep_(*E*), the probabilities that the free EcR is in its activating and repressing form, respectively, as a function of the concentration of 20E (denoted as *E*). With these simplifications, we then derived an expression for the normalized transcriptional activity *A* of a gene regulated by a single EcR and an additional constitutive transcriptional activator or repressor:

(Equation 1) $$\begin{array}{c} \frac{A(E)}{A_{\max}}=\frac{\chi (E)}{C_{\text{EA}}}\frac{1+\kappa_{p}C_{\text{T}}C_{\text{EA}}}{1+\kappa_{p}C_{\text{T}}\chi (E)}\\ \chi (E)=1+\left(C_{\text{ER}}-1\right)P_{\text{rep}}(E)+\left(C_{\text{EA}}-1\right)P_{\text{act}}(E). \end{array}$$

Here, the cooperativity coefficients (*C*_ER_, *C*_EA_, *C*_T_), modulate the affinity *κ*_*p*_ of the TM for the promoter, taking into account the configuration of EcR, i.e., in its repressive form (*C*_ER_ < 1), activating form (*C*_EA_ > 1) or neutral form, and/or the effects of the constitutive activator (*C*_T_ > 1) or repressor (*C*_T_ < 1). The function *A*(*E*) in Equation 1 is characterized by a finite baseline value that increases with *C*_T_ (Figure 4G; Methods S1). By adjusting *C*_T_ (the strength of the constitutive activator/repressor), the model can reproduce the characteristic normalized response of cluster 1, 2, and 3 genes (Figure 4G), which should be understood as arbitrary subdivisions of a continuum spectrum of responses determined by the parameter *C*_T_. Increasing *C*_T_ indeed reduces the overall fold change of activation, defined as the ratio of expression levels at high and low 20E concentration (Figures 4G and 4H). Across the values of *C*_T_, the predicted overall fold activation correlated positively with the predicted δ_(2,000-20)_/δ_(20-0)_ ratio (Figure 4H), as observed with real 20E target genes (Figure 4E). This correlation arises in the thermodynamic model through the dual effect of the constitutive enhancer: the enhancer indeed reduces the overall fold change of activation and also increases the effective affinity of 20E for DNA-bound EcR (compared to free EcR). This is because the enhancer stabilizes the TM at the promoter, which in turn thermodynamically favors 20E binding to DNA-bound EcR in the same way that 20E-EcR favors TM recruitment to the promoter (Methods S1). Conversely, silencers are predicted to increase the overall fold change of gene expression and increase the threshold of activation by 20E to a higher 20E concentration (Figure 4H). This predicted dual effect of constitutive enhancers and silencers provides a simple explanation for the correlation between the fold change increase of gene expression in response to 20E and their threshold of activation (Figure 4E). Overall, our mathematical analysis shows that combinations of constitutive enhancers and silencers with EREs can account for the complete range of responses of EcR target genes, even though one cannot be sure that such simple interactions generate the spectrum of authentic target gene responses (see discussion).

**Figure 5:**
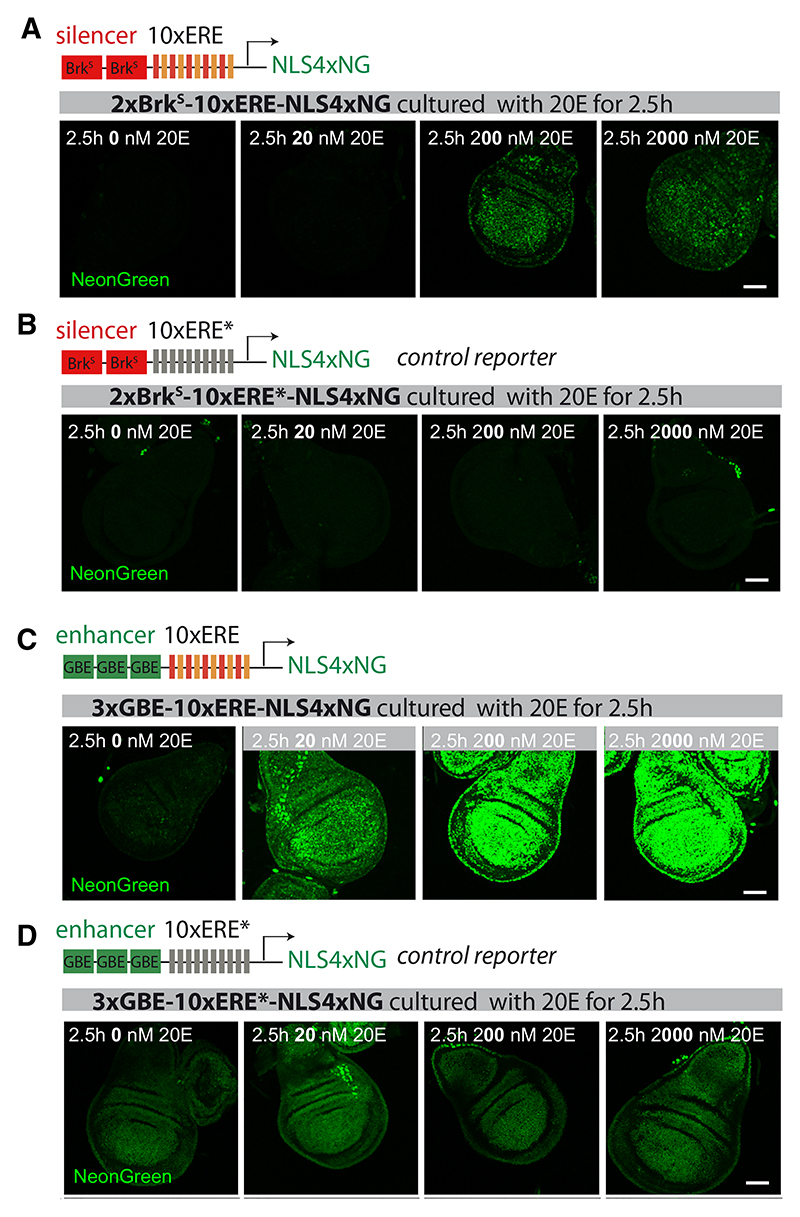
Emulation of various responses to 20E in synthetic reporters (A and B) Addition of a silencer (2xBrk^S^) raises the concentration of 20E needed to trigger activation by ERE. Although 20 nM 20E suffice for detectable activation of 10xERE-NLS4xNG (see Figure 2B), 200 nM are needed to activate 2xBrk^S^-10xERE-NLS4xNG. Therefore, this reporter emulates a high-threshold target gene. A control reporter with mutated ERE is not activated at any concentration (n ≥ 5 for each of the conditions) (B). (C and D) Addition of an enhancer (3xGRE) to 10xERE-NLS4xNG raises baseline activity, leading to weak, albeit detectable, signal even in the absence of 20E. Note that in the absence of 20E, the reporter is less active than the control reporter (mutated ERE) because of repression by unliganded EcR (n ≥ 5 for each of the conditions). The 3xGBE-10xERE-NLS4xNG reporter emulates the expression expected from pro-proliferative genes (responding to all physiological concentrations of 20E). Scale bars represent 50 μm.

We next proceeded to build synthetic reporters based on the principles outlined above. To mimic high-threshold target genes, we inserted upstream of 10xERE two copies of a silencer element from the *brinker* gene, Brk^S^,^63^ which has been shown to mediate constitutive repression in the prospective wing (schematic in Figure 5A). Activity of the resulting 2xBrk^S^-10xERE-NLS4xNG reporter was first assessed in transgenic imaginal discs at different developmental stages (Figures S6A and S6B). A fluorescence signal could only be detected at the end of the 3^rd^ instar, at the time of pupariation, suggesting that this reporter only responds to high-level 20E. A control transgene comprising mutated EREs (EREs*) was silent at all stages (Figure S6B), confirming that this reporter’s response to high 20E levels depends on functional EREs. We next assessed the activity of 2xBrk^S^-10xERE-NLS4xNG in cultured mid 3^rd^ instar imaginal discs treated with 20E at different concentrations. A fluorescence signal was only detected after treatment with high 20E concentrations (200 nM or more), whereas the control transgene had no activity, even at high 20E concentration (Figures 5A and 5B). Recall that a much lower concentration of 20E (20 nM) suffices to activate the simple 10xERE-NLS4xNG reporter (Figure 2D). We conclude therefore that addition of a constitutive silencer raises the activation threshold of a gene regulated by minimal EREs, pushing it toward the upper-right-hand side of cluster 3 in the response map.

To mimic genes located at the other end of the map (expressed across the range of 20E concentrations), we combined EREs with Grainy head binding elements (3xGBE), which confer constitutive enhancer activity in the prospective wing.^64^ In cultured mid 3^rd^ instar discs, the resulting reporter was expressed in a 20E concentration-dependent manner but with non-zero activity at 0 nM 20E (Figure 5C), as seen with cluster 1 genes. The corresponding control transgene (with ERE*) was expressed at an intermediate constitutive level at all concentrations of 20E (Figure 5D), as expected. At 0 nM 20E, this constitutive signal was higher than that of 3xGBE-10xERE-NLS4xNG (with wild-type EREs), indicating that, in the absence of 20E, EcR suppresses the activity of the constitutive enhancer. At 20 nM and above, the situation is reversed with the GBE-ERE combination overtaking the constitutive enhancer (GBE only) (Figure 5D; Methods S1). As an aside, we took advantage of 3xGBE-10xERE-NLS4xNG’s wide dynamic range to document the effect of β3-octopamine receptor (Figures S6C–S6F). Since 20E rises with larval age, we expect 3xGBE-10xERE-NLS4xNG’s activity to evolve accordingly as developmental time progresses beyond the L2-L3 transition (Figure S6G). Expression of this reporter at 24 h AL2-L3, but not at 44 h AL2-L3, is below the activity from the constitutive enhancer, highlighting once again the contribution of 20E in relieving the default growth-repressing activity of EcR during the growth phase of imaginal discs. Overall, the above results indicate that 3xGBE-10xERE-NLS4xNG emulates relatively well the behavior of low-level 20E target genes.

**Figure 6:**
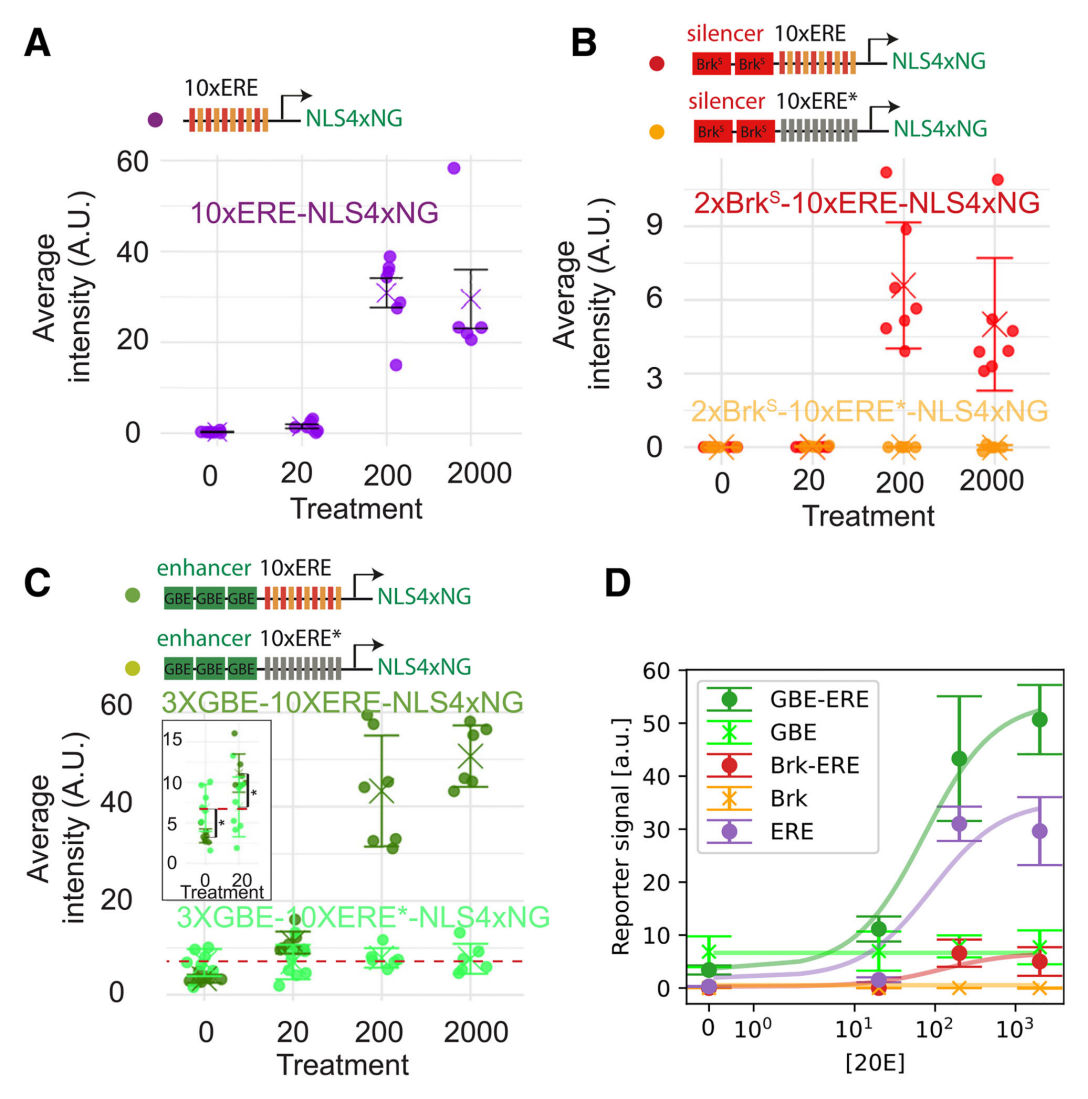
The thermodynamic model predicts cooperativity between EcR and the constitutive activator (A–C) Quantification of reporter activity (NeonGreen fluorescence) in transgenic wing discs cultured for 2.5 h at different concentrations (nM) of 20E (n ≥ 5 for each of the conditions). Error bars represent standard deviation. t tests were performed in (C). * p < 0.05. (D) Experimental data from (A)–(C) and fitted curves from the thermodynamic model for different constructs, assuming for simplicity that the activation probability of the set of EREs as a function of ecdysone equals that of a single ERE (more detailed descriptions accounting for interactions among EREs are explored in Figure S5F). Ecdysone levels are plotted on a symmetric logarithmic scale. Parameters: *κ_P_* ≃ 0.03, ${\bar{\kappa }}_{R}≃1$, *C_ER_* ≃ 0.1, *C_EA_* ≃ 13, *C_TA_* ≃ 2, *k_T_* ≃ 131 a.u(see Methods S1).

We then compared the experimental responses of the three transgenes (Figures 6A–6C) to the predictions of our thermodynamic model, using a numerical fit that allowed us to extract its free parameters (Figure 6D). The results showed that all response curves could be explained by the thermodynamic model. The best-fit parameters revealed that the enhancer-bound constitutive activator has a weaker effect on transcription (*C*_TA≃_2) than 20E-bound EcR (*C*_EA≃_13), indicating that EcR strongly activates transcription at high 20E concentration (see details in the Methods S1). In combination with the low basal affinity of the TM for the promoter (*k_P_* ≪ 1), this leads to a large overall fold change in response to 20E. Model fitting also implies that EcR acts as strong repressor at low 20E concentration (*C*_ER ≃_ 0:1) and that this repression is lifted at an 20E concentration of ~5 nM, broadly consistent with the observation that low 20E concentrations of 10, 20 nM can restore proliferation (Figures 3A and 3B). We found that the transgene response curves were best explained by models involving cooperativity between several EcR-bound ERE sites. However, to implement cooperativity, additional assumptions regarding the relative strength of the EREs and the “rules” of cooperation were needed, thus adding parameters and hence reducing the model’s predictive power (see details in Figures S5D–S5F and Methods S1). Nevertheless, our simplified theoretical results, along with the *in vivo* behavior of synthetic reporters, show that simple rules can reproduce the behavior of a wide range of EcR target genes. It remains to be seen to what extent natural target genes rely on such rules.

## Discussion

In this paper, we reconcile two seemingly opposite views on the effect of systemic 20E on the control of tissue proliferation. We show that, during the growth phase of imaginal discs, unliganded EcR acts as a brake to proliferation and that this brake is progressively released and overcome by the rising level of 20E. At much higher concentrations, such as those that are present at pupariation, 20E does not merely derepress EcR but, in addition, activates a gene expression program that triggers morphogenesis and proliferation arrest. The non-zero signaling activity seen in the absence of ligand and receptor is reminiscent of other signaling pathways whereby removal of the main transcriptional mediator leads to weak but significant ligand-independent signaling activity (e.g., T-cell factor [TCF] for Wnt signaling or Gli for Hedgehog signaling).^60,65–68^ This arrangement achieves a greater dynamic range by reducing signaling activity in the absence of ligand. In the case of EcR, it appears that three functional outputs are generated: (1) the absence of ligand prevents proliferation, (2) low ligand levels mimic the default pro-proliferative state (same as in the absence of EcR), and (3) additional signaling due to higher ligand levels terminates proliferation. Thus, depending on its systemic concentration, the same hormone has opposite effects on proliferation.

**Figure 7:**
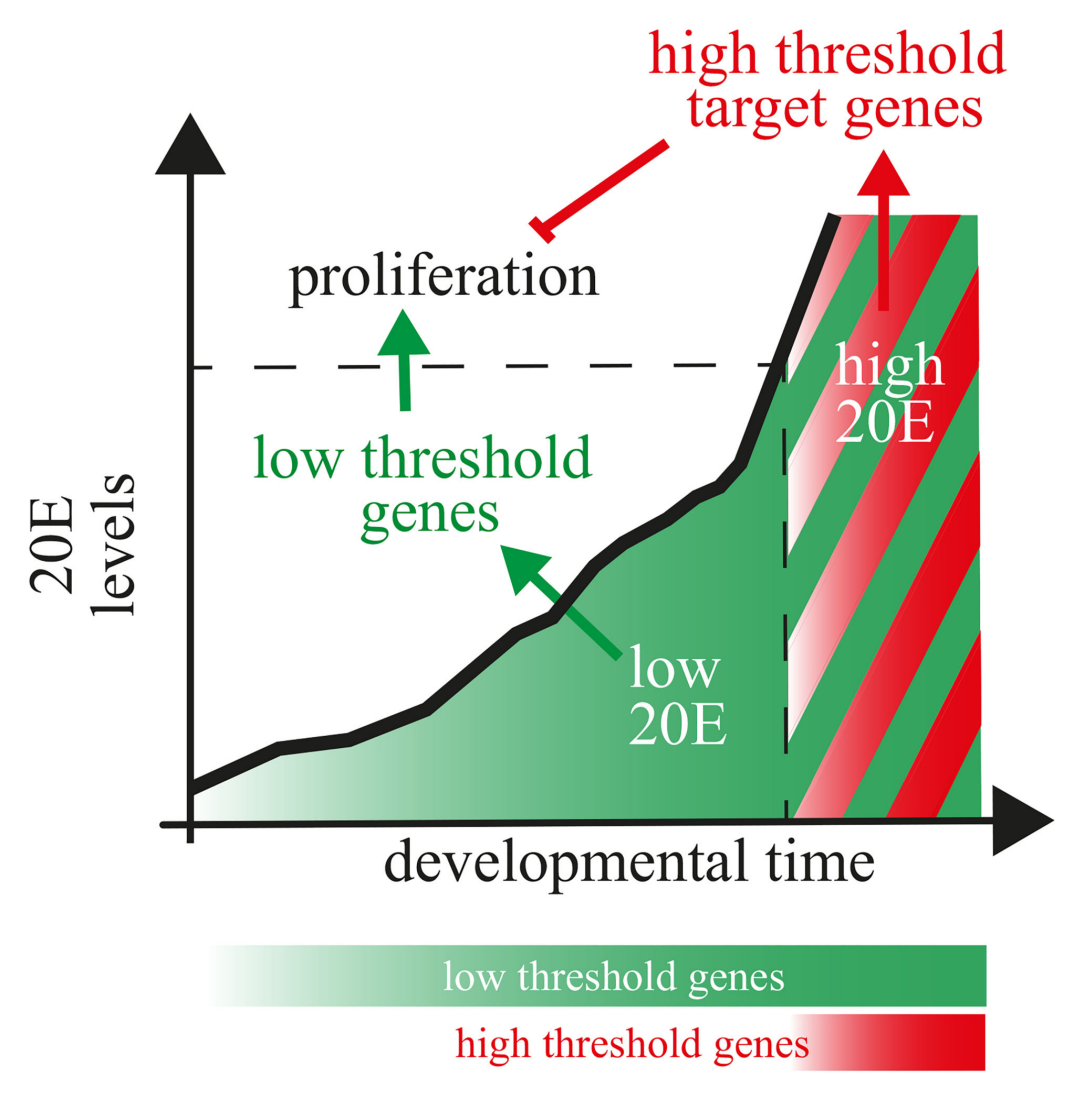
Model: control of cell proliferation by different levels of 20E Pro-proliferative genes increase in response to all levels of 20E, whereas highthreshold target genes (anti-proliferative) are only activated at high 20E concentrations. High-threshold anti-proliferative genes are proposed to dominantly suppress the activity of pro-proliferative genes, making this regulatory circuit an incoherent feedforward loop. We suggest that the presence of a constitutive enhancer or silencer modulates the activity of EREs. The repressive function of unliganded EcR would guarantee the inhibition of target genes in the absence of 20E, perhaps explaining the requirement of 20E for growth.

To further explore the dose-dependent activity of 20E, we turned to RNA-seq analysis of explanted imaginal discs exposed to a range of concentrations. Many genes were found to be activated in a dose-dependent manner, but they differed in the shape of their response curve. Some genes were primarily expressed at high 20E concentrations (cluster 3), whereas others were expressed across the entire range of physiological concentrations (clusters 1 and 2). Among the former, we found many genes previously shown to be activated at pupariation, when 20E levels are relatively high, e.g., Blimp-1, ImpE1, ImpE2, and ImpL2,^69^ validating our analysis. Genes involved in proliferation arrest are likely to be found among these high-threshold genes. However, so far, we have not been able to identify a gene that, on its own, is sufficient to prevent proliferation upon overexpression at mid 3^rd^ instar, presumably because proliferation arrest requires the coordinated activation of multiple high-threshold genes. At the other end of the spectrum, among genes expressed at low 20E concentrations, were *wg, dpp, hh*, and *Egfr*, which are required for imaginal tissue growth. These and also genes involved in Hippo and mTor signaling have been shown previously to be regulated by 20E^27–30^ either directly, as suggested by EcR chromatin immunoprecipitation sequencing (ChIP-seq) analysis^47^ or indirectly via modulation of Taiman (Tai), an EcR cofactor.^70,71^ We note, however, that these genes are expressed in the absence of 20E, when proliferation is not sustained. Perhaps they only stimulate proliferation in concert and over a combined threshold. Alternatively, additional 20E-responsive genes not uncovered by our analysis (perhaps because they are not directly activated by 20E) may be required. It is worth pointing out that none of the 1,489 genes identified as modulated by 20E (listed in Table S1) were expressed only in the pro-growth concentration range (i.e., not expressed at the high 20E levels that trigger proliferation arrest). We suggest, therefore, that the anti-proliferative genes that are expressed at high concentration must override the effect of pro-growth target genes. Thus, growth could be regulated by an incoherent feed-forward loop that inverts the effect of 20E at high concentration (see diagram in Figure 7).

To understand how a simple regulatory element can achieve qualitatively distinct dose responses, we took a synthetic approach, first *in silico* and then *in vivo*. Our results suggest that a combination of relatively simple regulatory elements can account for the range of response to various 20E concentrations. Since 20E concentration increases over time during development, these responses can in principle give rise to various patterns of temporal activation of genes, analogous to spatial morphogen gradients defining spatial domains of target gene expression (Figure S5E^62^). However, other features are also likely to be relevant. For example, McKay and colleagues have shown that chromatin accessibility to EREs, which varies between tissues and over developmental time, is a key determinant of the EcR’s response.^72^ Moreover, several targets of 20E signaling are known to modulate EcR activity (for example, Eip78C^73^), highlighting the importance of feedback control and the integration of signals over time. These features could be incorporated in an expanded model that makes the activity of the constitutive enhancer or of liganded EcR dependent on current or historical 20E levels. Nevertheless, in its current form, our model shows that a small number of regulatory elements can account for the spectrum of responses to 20E, at least at a given developmental stage.

In summary, our work explains how a given type II nuclear receptor can drive opposite cellular responses depending on the levels of its ligand. We showed that the presence of a constitutive enhancer or silencer could determine whether a target gene responds only to high hormone concentrations or to a broader range that also includes low concentrations. We further suggest that a bimodal response can be achieved if the high-threshold genes suppress the activity of the low-threshold genes, a mode of regulation that could also be relevant to vertebrate type II nuclear receptors such as the retinoic acid receptor^74,75^or the thyroid receptor.^76,77^ Moreover, our work highlights the possibility that inactivation of the receptor, e.g., with a chemical degrader, may not achieve the same objective as hormone depletion.

### Limitations of the study

Our *ex vivo* RNA-seq analysis identifies a list of 20E target genes that respond to different concentration of 20E. However, further genetic analysis will be needed to identify the specific target genes that mediate the effects of this hormone on proliferation. We expect that the genes mediating proliferation arrest will be found among the high-threshold targets. One such gene encodes Blimp-1, although preliminary gain- and loss-of-function experiments show that it is unlikely, on its own, to control proliferation arrest. We suggest that proliferation arrest may require the combined activity of multiple high-threshold target genes. Their identification will require systematic combinatorial gain-of-function analysis. Identification of all the relevant targets will also enable to test whether, as suggested by our *silico* analysis and the behavior of our synthetic reporters, a simple incoherent feedback module does underpin the bimodal effect of 20E on proliferation.

## Star⋆Methods

Detailed methods are provided in the online version of this paper and include the following:

- KEY RESOURCES TABLE
- RESOURCE AVAILABILITY
  ○ Lead contact
  ○ Materials availability
  ○ Data and code availability
- EXPERIMENTAL MODEL AND STUDY PARTICIPANT DETAILS
  ○ Fly husbandry
- METHOD DETAILS
  ○ Developmental curves
  ○ Wing disc culture and imaging
  ○ Pupal imaging
  ○ Volumetric analysis
  ○ Molecular biology and cloning
  ○ Sample preparation for RNA-seq
  ○ RNA-seq analysis
  ○ Mathematical simulations
- QUANTIFICATION AND STATISTICAL ANALYSIS

## Star⋆Methods

### Key Resources Table

**Table:** 

REAGENT or RESOURCE	SOURCE	IDENTIFIER
Antibodies		
anti-GFP	Abcam	RRID: AB_300798
Anti-chick-488	Life tech	RRID: AB_2534096
Critical commercial assays		
FocusClear™	2BScientific	FC-101
MountClear™	2BScientific	MC-301
Vectashield ®	Vector labs	H-1200-10
Deposited data		
RNA sequencing data	This paper	GEO: GSE236166
Experimental models: Organisms/strains		
*D. Melanogaster phtm-Gal4 UAS-GFP*	Federica Mangione / Nic Tapon lab (The Francis Crick Institute)	N/A
*D. Melanogaster pdm2^R11F0^2-Gal4*	Bloomington	49828
*D. Melanogaster nub-Gal4*	Bloomington	86108
*D.Melanogaster UAS-Octβ3R^RN^‘^Ai^*	Bloomington	31108
*D.Melanogaster EcR-GFP°^KO^*	This work	N/A
*D.Melanogaster EcR^KO^*	This work	N/A
*D.Melanogaster UAS-Flp*	Bloomington	4540
*D.Melanogaster LexOP-Flp*	Bloomington	55819
*D.Melanogaster rotund-LexA*	This work	N/A
*D.Melanogaster 10xERE-4xNG*	This work	N/A
*D.Melanogaster 2xBrkS-10xERE-NLS4xNG*	This work	N/A
D.Melanogaster 2xBrkS -10xERE*-NLS4xNG	This work	N/A
*D.Melanogaster 3GBE-10xERE-NLS4xNG*	This work	N/A
D.Melanogaster 3GBE-10xERE*-NLS4xNG	This work	N/A
*D.Melanogaster UAS-EcR^RNAi^*	Bloomington	9327
*D.Melanogaster Vg-Gal4 UAS-Flp tub-FRT-STOP-FRT-Gal4*	Crickmore and Mann^78^	N/A
*D.Melanogaster Br-Z1-GFP*	Bloomington	50754
*D.Melanogaster Blimp-1-GFP*	Bloomington	67656
*D.Melanogaster His2AV-mRFP*	Morillo Prado et al.^79^	N/A
*D.Melanogaster E-Cad-GFP*	Huang et al.^80^	N/A
Software and algorithms		
Fiji v. 2.0.0/1.53t	Schindelin et al.^81^	RRID: SCR_002285
RStudio 2022.07.0+548	R Development Core Team^82^	RRID: SCR_000432
Wolfram Mathematica v. 12.3	Woldfram Research^83^	RRID:SCR_014448
Python v. 3.9.7	Van Rossum and Drake^84^	RRID:SCR_008394
Matplotlib v. 3.5.0	N/A	RRID:SCR_008624
HISAT2 v. 2.1.0	Kim et al.^85^	RRID:SCR_015530
SAMtools v.1.13	Li et al.^86^	RRID:SCR_002105
Subread v. 1.6.4	Liao et al.^87^	RRID:SCR_009803
SLURM	Yoo et al.^88^	https://slurm.schedmd.com/quickstart.html
DESeq2	Love et al.^89^	RRID:SCR_015687
R cluster package v. 2.1.4	Maechler et al.^90^	https://cran.r-project.org/web/packages/cluster/index.html
Cytoscape v. 3.9.1	Shannon et al.^91^	RRID:SCR_003032
Iregulon v.1.3	Janky et al.^92^	http://iregulon.aertslab.org
Custom Code for Modeling	This work	https://doi.org/10.5281/zenodo.8279944

### Resource Availability

#### Lead contact

Further information and requests for resources and reagents should be directed to and will be fulfilled by the lead contact (jp.vincent@ crick.ac.uk, perezg@crick.ac.uk, guillaume.salbreux@unige.ch, luca.cocconi@ds.mpg.de).

#### Materials availability

Flies and plasmids are available from the lead contact.

#### Data and code availability

- The codes of the simulations are available on the GitHub repository: https://github.com/lucocconi/EcR_transcription
- The RNA-seq data is publicly available at the Gene Expression Omnibus: GSE236166.
- Any additional information required to reanalyze the data reported in this paper is available from the lead contact upon request.

## Experimental Model And Study Participant Details

### Fly husbandry

Flies were raised in food containing 6 g/l Agar, 30 g/L Wheat flour, 70 g/L dried yeast, 50 g/L Glucose, 1.95 g/L Nigapen and 7.8 mg/L Bavistan. The animals were kept at 25C in a Sanyo incubator with 12h light/dark cycles.

## Method Details

### Developmental curves

Flies were allowed to lay eggs for 4 h intervals between 8:00 and 20:00. 2 x 30 L1s for each of these three plates were then transferred to fly vials (six tubes in total) and pupae formation was scored every 4 h between 8:00 and 20:00.

### Wing disc culture and imaging

Dye medium was prepared as described in Dye et al.^29^ and Dye.^93^ Briefly, Grace’s medium (Sigma, G9771) containing 5mM BisTris had its pH adjusted to 6.6-6.7. Prior to each experiment, it was supplemented with 5% FBS (ThermoFisher/Invitrogen, 10270098), 1x Pen/Strep (Sigma P4333, 100x stock solution) and different concentrations of 20E (Sigma, H5142).

Live imaging experiments were performed by mounting wing discs in an uncoated ibidi 35mm imaging dish (Ibidi, 81141) as described in Hecht et al.^37^ We used a Nikon CSU-W1 Spinning Disk, to image the disc every 5 min with a Z-interval of 0.75 μm and using the 60x objective.

To quantify proliferation, a max projection of the movies’ frames was first performed. Cells undergoing mitosis were manually tracked within ROIs of similar areas for the various conditions and replicates. Mitotic cells were identified as either undergoing mitotic rounding (when using E-Cad-GFP) or chromosome segregation (when using His2AV-mRFP), using a custom FIJI code. The number of mitotic events was then averaged within a 1 h rolling window to calculate the number of mitoses.

For ex vivo culture, 24 h AL2-L3 discs were dissected and incubated for 2.5h at 25 °C in Dye medium supplemented with different 20E levels. They were then fixed in 4% formaldehyde (Pierce 28906) for 45 min.

For the Br-Z1-GFP and Blimp-1-GFP experiments, the discs were staining with anti-GFP (Abcam ab13970 1:500), and Anti-chick-488 (Life tech A11039, 1:1000).

For NeonGreen fluorescence, Br-Z1-GFP and Blimp-1-GFP immunofluorescence quantifications a custom code was used to calculate the intensity of the signal inside the nucleus (marked with DAPI) minus the noise measured outside of the nucleus.

Data presented in Figure S6 was used to normalize data from the 10xERE-NLS4xNG and the 3xGBE-10xERE-NLS4xNG/3xGBE-10xERE*-NLS4xNG/2Brk^S^-10xERE-NLS4xNG/2Brk^S^-10xERE*-NLS4xNG reporters, which were acquired under different conditions. Expression of Br-Z1 and Blimp-1 proteins was inferred from staining knock-in strains with chicken anti-GFP (Abcam Ab139701:500) and an anti-chicken secondary antibody (Invitrogen A-21437 1:1000).

### Pupal imaging

A single focal plane was recorded every 20 min on a live cell imaging Nikon LTTL 1 (4x objective). For quantification, an ROI was manually selected as shown in the Videos S2 and S3, and the average fluorescence intensity was measured inside this ROI at every time point.

### Volumetric analysis

Volume quantifications were obtained from staged wing discs fixed for 45 min in 4% PFA (Pierce 28906), stained with Vectashield® (Vector labs, H-1200-10), mounted in agar as described in Hecht et al.,^37^ and imaged with an upright Leica SP5 confocal microscope. Since Vectashield® DAPI non-specifically stained at low levels the whole wing imaginal disc tissue, it was possible to use Imaris to generate 3D reconstructions and measure tissue volume.

### Molecular biology and cloning

The EcR^*cKO*^ line was generated by removing the last four exons common to all EcR isoforms and replacing them with an attP sequence. The two CRISPR target sites used were located 526 bp upstream of the four last common exons, and 1 bp after the stop codon in the last exon. We then re-inserted a genomic fragment containing the last four exons, eGFP before the stop codon and 2 kb of 3’UTR.

*EcR*^KO^ was made by removing the last 3 exons using a CRISPR target site located 132 bp upstream of the last 3 exons, and another located 32 bp after the last exon.

For the various NeonGreen reporters, we used the GeneArt Gene Service from Thermo Fischer (https://www.thermofisher.com/uk/en/home/life-science/cloning/gene-synthesis/geneart-gene-synthesis.html) to synthetize either 3xGBE, 2xBrk^S^, 10xERE, or 10xERE*. The sequences were then inserted in the relevant order using restriction digest and ligation.

The *rotund-LexA* driver was generated by exchanging a MIMIC cassette (from the line BL44158) into a T2A-LexA cassette as described by Diao et al.^94^

All sequences and details are available upon request.

### Sample preparation for RNA-seq

24 h AL2-L3 wing discs were incubated for 2.5h at 25°C in Dye medium (see wing disc culture) supplemented with different concentrations of 20E (Sigma, H5142). Discs were then snap-frozen in dry ice. After all the samples were collected, RNA was extracted with a RNeasy Mini Kit (Qiagen, 74104). 1.2-2.1 μg were used as a template to generate a sequencing library with the NEBNext Ultra II Directional PolyA mRNA (NEB, E7760S). The Advanced Sequencing Facility of the Francis Crick institute used an Illumina HiSeq 4000 to perform single end 1 x 75 bp sequencing.

### RNA-seq analysis

The reads were aligned to BDGP6, reference genome (ensemble release 84), using HISAT2(v. 2.1.0).^85^ SAMtools (v. 1.13)^86^ allowed to first transform the HISAT2-generated SAM files into BAM files and then to sort and index them. FeaturesCounts from the package Subread (v. 1.6.4)^87^ was used with the options -t exon \ -g gene_id \ –primary to count reads mapping with features. All these tasks were parallelized using SLURM.^88^ Principal component analysis was performed on the vst transformed (option blind=TRUE) raw data, and plotted using the plot PCA function of DESeq2.^89^

DESeq2 was used to determine the genes that displayed a change in expression that could be explained by changes in ecdysone levels (Likelihood ratio test p adjusted value<0.001). From the resulting 1,489 genes (listed in Table S1), we studied the average counts we decided to ignore those expressed at low level (total average counts from the four experimental conditions below 500), deemed unlikely to have biological significance, and focused on those that change monotonically between 0, 20 and 200 nM. The average counts (normalized using DESeq2’s median of the ratio methods) were further normalized to the maximum value for each gene, and then separated into clusters using k-medoids clustering from the R cluster package (v. 2.1.4^90^). The optimal cluster number was determined using the elbow method on a graph representing the total intra-cluster variation in function of the number of clusters.

For the randomly generated data, we pooled the dataset containing all the (1,489) genes affected by ecdysone (Table 1). We then calculated the mean and standard deviation of expression levels across the whole dataset and used a gaussian distribution with the same mean and standard deviation to generate a dataset composed of 50 000 artificial genes. Those with negative values of expression were excluded and the same filtering used previously to select the upregulated genes was then used to filter down this randomly generated dataset. This led to the 8359 artificial genes displayed in the Figure S4J.

The Cytoscape’s (v. 3.9.1)^91^ plugin Iregulon (v.1.3)^92,95^ was used to detect TF motif enrichment in the regulatory region of genes of interest. This algorithm ranks motifs using a NES score which represents a statistical assessment of enrichment (more details in Janky et al.^92^). As many transcription factors (TFs) bind motifs that have a certain degree of similarity, they were grouped in *clusters*. The table in Figure S4 presents the five most enriched clusters in the two datasets. #Motifs shows the number of motifs that are recognized by the members of a given TF cluster. #Targets show the number of genes that contain binding motifs recognized by the TFs of a given cluster. The following parameters were used: motif collection, 10K; species and gene nomenclature, *Drosophila melanogaster*; Flybase names; region-based specific parameters, Overlap fraction 0.4, 10kb upstream, full transcript and 10kb downstream; recovery enrichment score threshold, 2.5; ROC threshold, 0.001; rank threshold 5000, TF prediction minimum identity, 0.0; FDR, 0.001.

### Mathematical simulations

Simulations were performed according to the model and method described in Methods S1.

## Quantification And Statistical Analysis

Sample number (number of wing discs, or cells), statistical significance (* for p<0.05, ** for p<0.01 and *** for p<0.001) and dispersion measures (standard deviation or standard error) appear in the figures and figure legends. The statistical tests were performed using Rstudio.

For Figures 1, 1B, 1D, 1F, 6C, and S1H normality was first tested using a Shapiro-Wilk test and then either a Wilcoxon signed rank sum test or a t-test was used.

## Supplementary Material

Supplemental information

Table S1

Table S2

Video S1

Video S2

Video S3

## Highlights

- The steroid hormone 20E has a bimodal effect on proliferation
- Low-level 20E abrogates the default anti-proliferation activity of the 20E receptor
- An additional transcription program activated by high 20E suppresses proliferation
- Simple regulatory elements suffice to recapitulate the bimodal activity of 20E
