# Supplementary material for: The Drosophila ecdysone receptor promotes or suppresses proliferation according to ligand level
Source: Dev Cell. Author manuscript; Available in PMC 2024 Feb 21. (PMC7615657; doi:10.1016/j.devcel.2023.08.032)
Supplement: Supplemental information [file EMS193946-supplement-Supplemental_information.pdf]

Developmental Cell, Volume *58*

Supplemental information

The *Drosophila* ecdysone receptor promotes or suppresses proliferation according to ligand level

Gantas Perez-Mockus, Luca Cocconi, Cyrille Alexandre, Birgit Aerne, Guillaume Salbreux, and Jean-Paul Vincent

Supplementary Figure 1

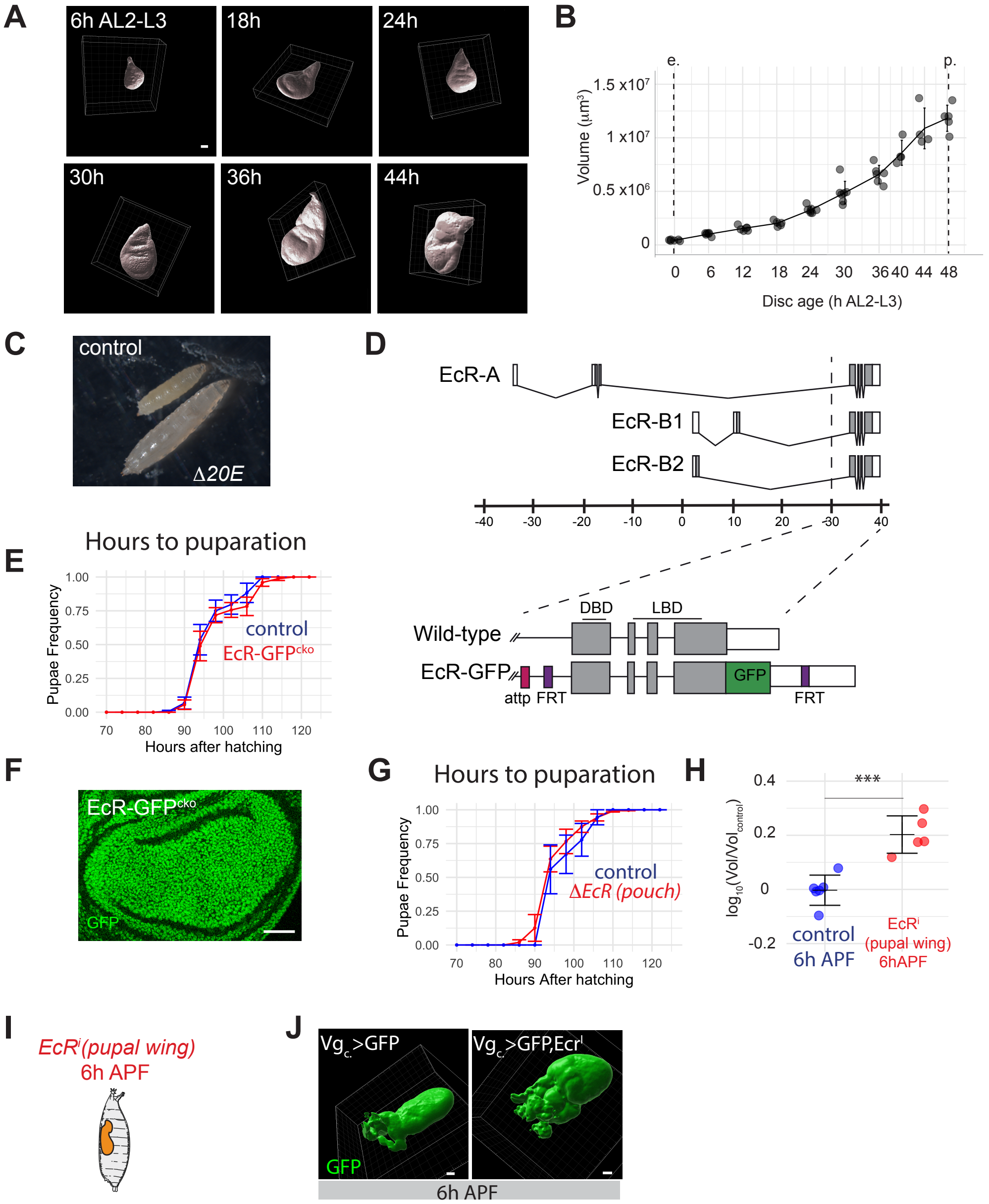

Sup. Fig.1 related to Figure 1. Wing growth and genetic tools to assess role of EcR.

A. Representative images of the volumetric reconstruction of L3 wing discs and pupal wings. **B.** Volume quantification during growth (n≥5 wing disc for each timepoint, except for 40 and 44h AL2-L3 where n=4). **C.** When 20E production is impaired (Δ20E*; phtm>Octβ3*^{i}), larvae do not pupariate and continue growing. **D.** Schematic representation of the EcR locus showing the three isoforms A, B1 and B2. In EcR-GFP^{cKO}. A GFP tag was inserted as shown and FRT sites were inserted to flank the last four exons, which are common to all isoforms. **E**. Data showing that EcR-GFP^{cKO}, which is homozygous viable does not affect developmental timing (n≥30 animals). **F.** The product of EcR-GFP^{cKO}, detected by GFP fluorescence, localizes to the nucleus. **G.** Inactivation of EcR-GFP^{cKO} (*pdm2>FLP EcR-GFP*^{cKO} */ EcR*^{KO}) had no impact on developmental timing (n≥22 animals). **H-J.** Whole wing knockdown of EcR in the whole wing with constitutive Vg-Gal4 driving an RNAi transgene (*Vg-Gal4 UAS-Flp Tub-FRT-STOP-FRT-Gal4/ UAS-EcR*^{RNAi}) leads to overgrowth, as measured 6h after puparation. See quantification in H (n≥5 for each of the conditions), experimental design in I and representative images in J. Error bars represent standard deviation. Scale bars represent 50 µm. Wilcoxon rank-sum statistic test for two samples was performed in D, I and L. * P<0.05, ** P<0.01 *** P<0.001. N.S. No statistical difference.

Supplementary Figure 2

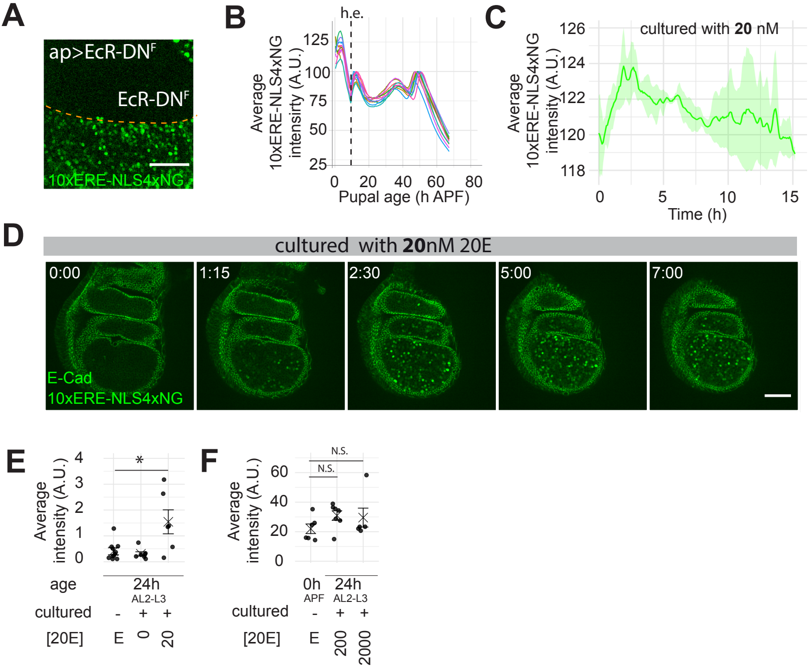

Sup. Fig.2 related to Figure 2. Validation of a minimal 20E responsive reporter **A.** Expression of a dominant negative form of EcR inhibits the 10xERE-NLS4xNeonGreeen reporter in wing imaginal discs. Expression is induced in the dorsal compartment with *ap-Gal4*, leaving the ventral compartment as control. **B.** Quantification of whole live larva fluorescence produced by the reporter around the time of pupariation. Each line represents the average NeonGreen signal for a single pupa. Dashed line marks head eversion (h.e.). hAPF = hours after pupal formation. **C-D.** Activation of the 10xERE-NLS4xNG reporter in transgenic explanted imaginal discs treated with 20nM 20E. Time after addition of 20E is indicated. Activation is seen within 2.5h. The quantification for several discs (n=3) is shown in B and representative images are shown in D. **E-F**. Quantification of reporter activity (NeonGreen fluorescence) in transgenic wing disc fixed directly after dissection or cultured for 2.5 h for different concentrations of 20E, expressed in nM. n≥5 for each of the conditions. Error bars represent standard deviation. Wilcoxon rank sum tests or T-tests were performed in C and D. * $p<0.05$ N.S. = no statistical difference. Error bars represent standard deviation. E refers to endogenous 20E. Scale bars represent 50 µm.

Supplementary Figure 3

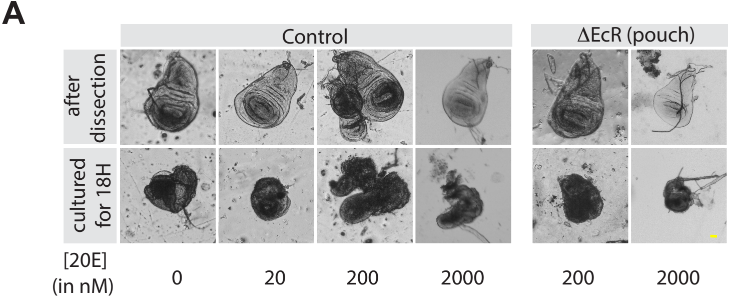

Sup. Fig.3 related to Figure 3. Role of EcR in disc eversion.

A. High concentration (200 or 2000nM) but not low concentration (20nM) of 20E triggers eversion (recognised as tissue elongation) in wild type mid 3rd instar imaginal discs after 18h of incubation. This does not occur if EcR is deleted from the pouch *ΔEcR; EcR-GFP^{cKO} pdm2-Gal4 UAS-Flp*). n≥5 for each of the conditions. Scale bar represents 50 μm.

Supplementary Figure 4

A

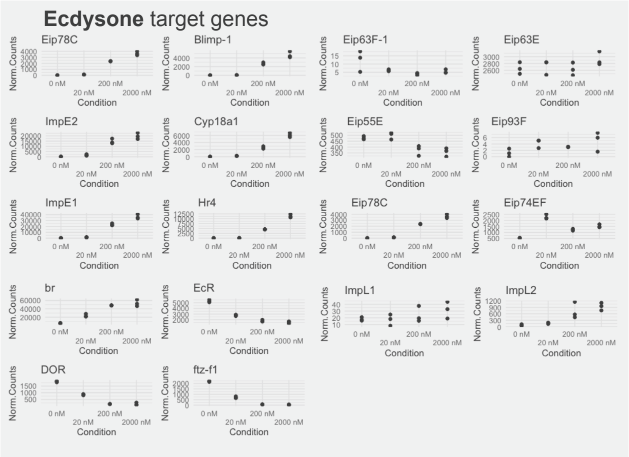

B

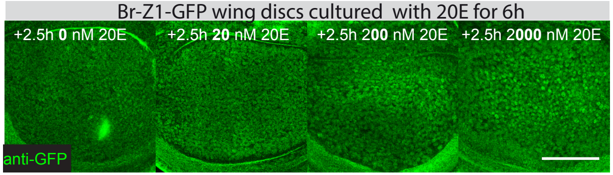

C

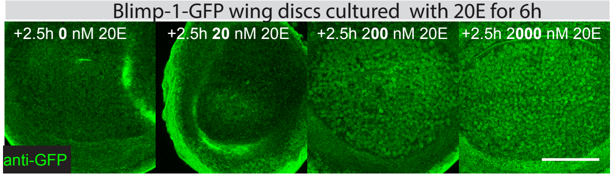

D

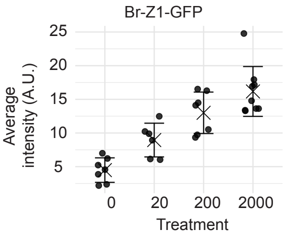

E

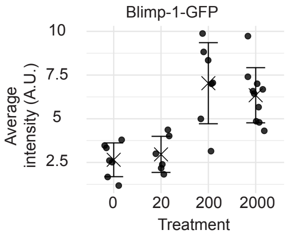

F

Top TF binding motifs enriched in **UPREGULATED** genes (611)

Cluster	TF	#Motifs	NES	#Targets
1	CG33260, Trl	15	5.260	347
**2**	**EcR**	**6**	**4.586**	**319**
3	ci, sug, lmd, opa	11	4.449	149
4	Med,pnr,pdm3,rn,br,cnc...	34	4.320	388
5	nau	4	4.192	340

G

Top TF binding motifs enriched in **DOWNREGULATED** genes (635)

Cluster	TF	#Motifs	NES	#Targets
1	gem, Myb, zh1, grh	9	4.807	300
2	Jra, Atff3, Xbp1, CrebB-17A...	33	4.679	305
3	CG33260, Trl	17	4.259	245
4	tra2	3	3.872	244
5	Top2	3	3.732	217

H

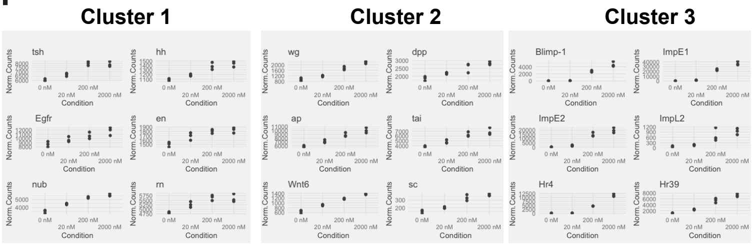

I

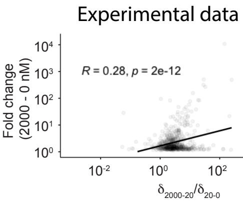

J

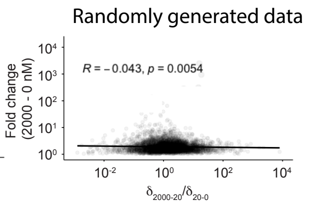

Sup. Fig.4 related to Figure 4. Expression of known 20E-regulated genes in explanted discs treated with various 20E concentrations.

A. Normalised reading counts for genes known to be affected (directly or indirectly) by 20E. **B-E** Levels of Br-Z1-GFP (n≥5) and Blimp1-GFP (n≥5), as assessed with anti-GFP, rise with increasing levels of 20E in explanted discs. **F**. Iregulon analysis[S1] was used to assess the enrichment of TF binding motifs in the vicinity of the genes increasing monotonically. As groups of TFs bind to highly similar motifs, the analysis groups them into clusters. For the upregulated genes, the canonical EcR binding site is the second most abundant TF binding motif present in the vicinity of the gene. NES score provides a statistical representation of enrichment[1]. #Motifs shows the number of motifs that are recognised by the members of a given TF cluster. #Targets show the number of genes that contain binding motifs recognised by the TFs of a given cluster. **G.** Downregulated genes are not enriched for EcR-binding motifs. **H**. Example of read counts for genes from each of the three clusters. **I, J**. The Pearson correlation coefficient between the fold change and the $\delta_{(2000-20)}/\delta_{(20-0)}$ ratio was calculated for the experimental data (611 genes up-regulated by ecdysone) (**I**) and for data randomly generated (see Material and Methods) (**J**). The two correlation coefficients differed statistically (Fisher's z-Tests for differences of correlations in two independent samples z = 7.81 with probability = 0). Error bars represent standard deviation, the scale bars represent 50 µm.

Supplementary Figure 5

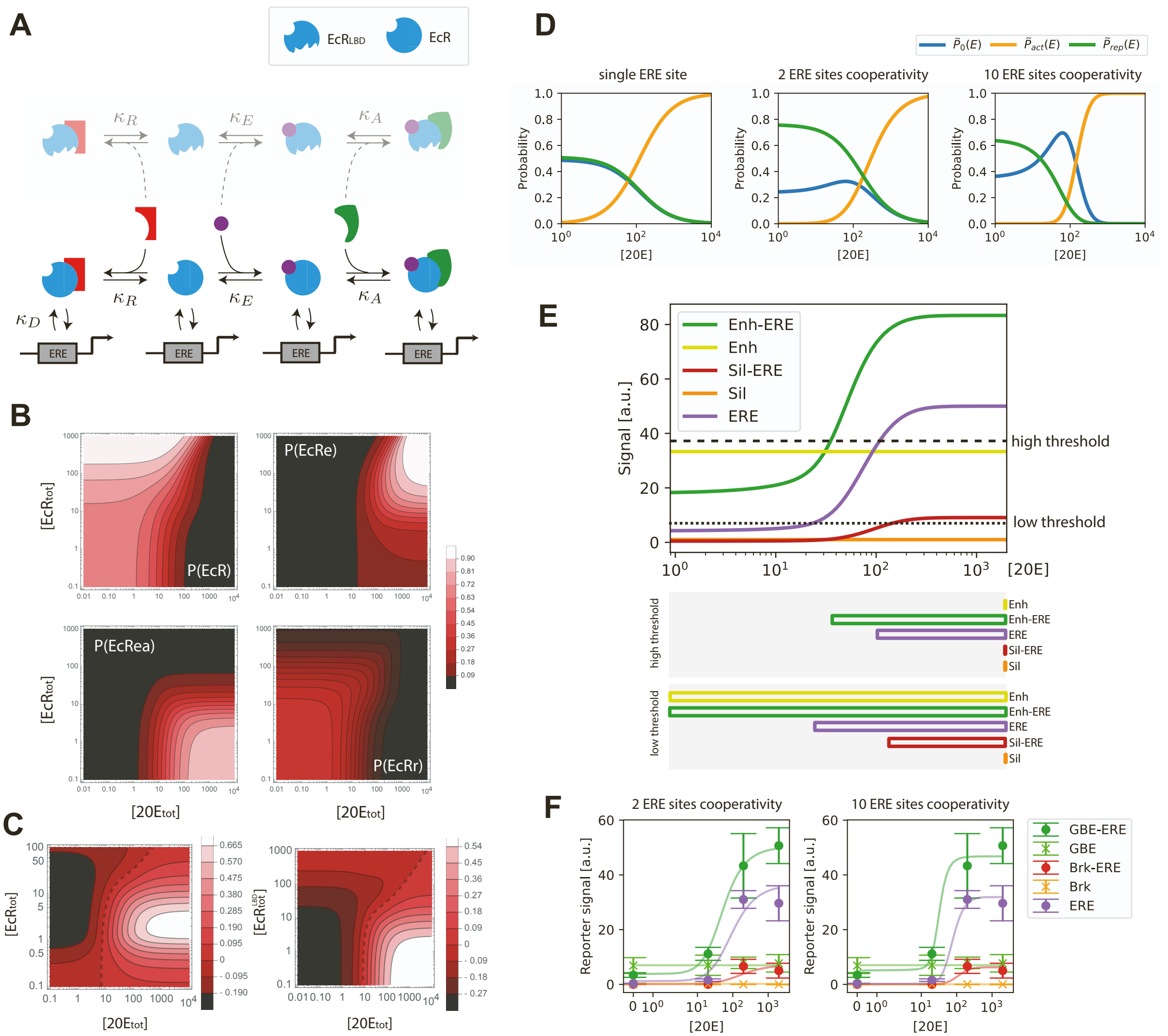

Sup. Fig.5 related to Figure 4. Models of gene regulation by Ecdysone.

A. Diagram representing the molecular complexes that EcR (blue) makes with 20E (purple dot), its coactivator (green), and corepressor (red). Arrows indicate reversible binding events with affinity specified by the ratio of *on* and *off* rates. We also consider a truncated form of EcR lacking a DNA binding domain (EcR^{LBD}), which acts as a "ligand sponge" to sequester ecdysone and other EcR binders. **B.** Probability for any given EcR to be found in each of the four accessible complexes described in panel A as a function of 20E and EcR concentration (in nM here and henceforth within this figure). The concentration of corepressor is assumed constant. As expected, EcR is mostly found in its activating form (EcRea) when EcR levels are low and 20E level are sufficiently high to expel the corepressor. Similarly, the repressive form of EcR (EcRr) is predominant in the low EcR, low 20E range. Parameters: $\kappa_A = 1 \text{ nM}^{-1}, \kappa_E = 0.017 \text{ nM}^{-1}, \kappa_R = 0.017 \text{ nM}^{-1}, A_{tot} = 5 \text{ nM}, R_{tot} = 30 \text{ nM}$, $ECR^{LBD}_{tot} = 0 \text{ nM}$. **C.** Visualisation of the context-dependent transcriptional action of a functional ERE, obtained by assigning a "modulation factor" to each EcR complex and computing a statistical average drawing on the probabilities of the ERE being occupied by EcR *and* the EcR being in a particular complex (see Methods S1 Mathematical Modeling). Left: When the sponge is not expressed ($EcR^{LBD}_{tot} = 0$), transcriptional activity is impaired by removing or overexpressing the EcR. Right: effect of various concentrations of EcD_{LBD} with EcR fixed at $ECR_{tot} = 5$ nM. Other parameters: $\kappa_A = 1 \text{ nM}^{-1}, \kappa_E = 0.017 \text{ nM}^{-1}, \kappa_R = 0.017 \text{ nM}^{-1}, A_{tot} = 5 \text{ nM}, R_{tot} = 30 \text{ nM}$, $\kappa_D = 20 \text{ nM}^{-1}$, $ERE_{tot} = 1$ nM. At low 20E, increasing EcR^{LBD} leads to derepression, while at high 20E levels it causes deactivation, recapitulating the results of[S2]. **D.** Probability of a set of EcRs effectively acting as transcriptional activator $\tilde{P}_{\text{act}}(E)$, transcriptional inhibitor $\tilde{P}_{\text{rep}}(E)$ or a neutral unliganded element $\tilde{P}_0(E)$ in three different models: left, one regulating EcR; middle, two regulating EcRs, right, 10 regulating EcRs. See Supplementary Informations for details and additional assumptions. Parameters: $\bar{\kappa}_R = 1.04$, $\kappa_E = 0.017 \text{ nM}^{-1}$; these are

kept constant to illustrate the effect of different models. **E.** Effect of enhancer and silencer elements on the activity of ERE. Addition of a silencer (orange curve) decreases the overall response of ERE (purple and red curves), while an enhancer (lime curve) enforces increased baseline activity (purple and green curves). Equivalently, the ERE modulates the enhancer's baseline activity by 20E-dependent switching from repressor to activator (lime and green curves). Silencers or enhancers modulate the concentration (and hence developmental time) at which high and low target genes cross a hypothetical threshold (black dashed and dotted curves, respectively), thus mimicking the mechanism that leads to nested expression of morphogen target genes. Parameters are those obtained through the fitting shown in panel F, except for C_{TA} which was increased to $C_{TA} = 10$ here for the sake of visual clarity. **F.** Fitting of the reporter activity as a function of ecdysone for different constructs (data shown in Fig. 6A-C) with the thermodynamic model using two alternative expressions for the probabilities $\tilde{P}_{\mathrm{act}}(E), \tilde{P}_{\mathrm{rep}}(E)$ and $\tilde{P}_0(E)$ (examples of which are plotted in panel D, middle and right). Cooperative effects among the EREs lead to a sharper overall response of gene expression as a function as ecdysone concentration compared to the model with only ERE (Fig. 6D), which is in better agreement with the experimental data. Error bars represent standard deviation. See Methods S1 Mathematical Modeling in Material and Methods for fitted parameters.

Supplementary Figure 6

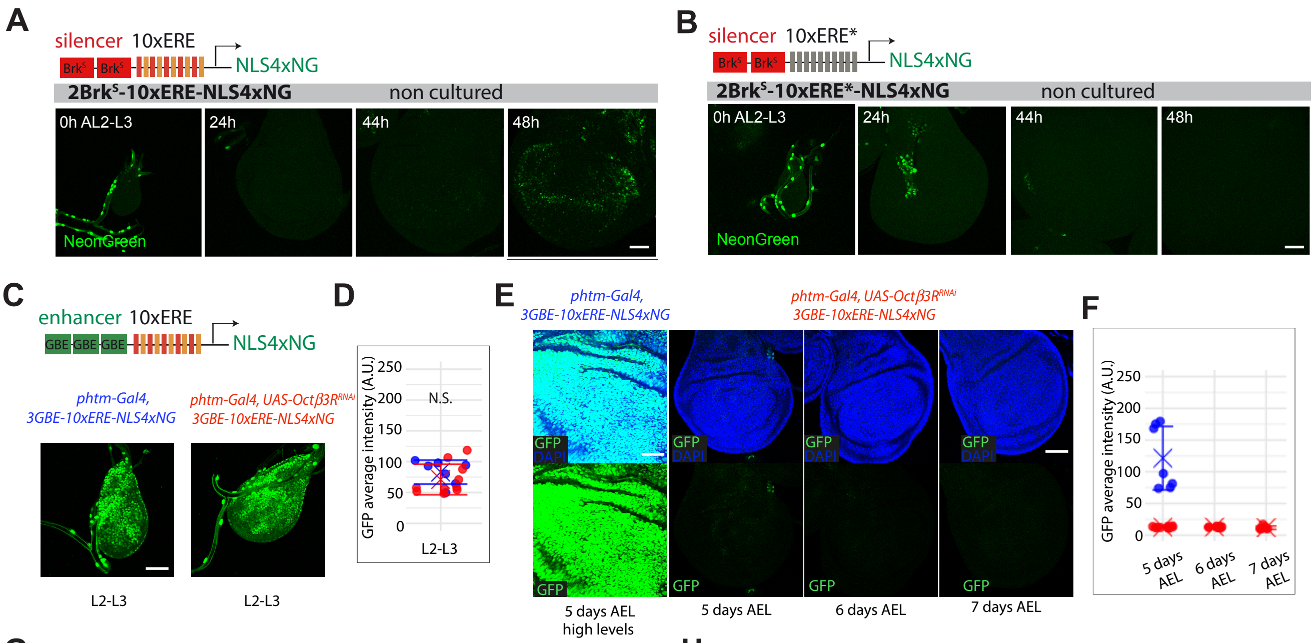

Sup. Fig.6 related to Figure 5. Expression of the four synthetic reporters in freshly explanted discs.

A. 2xBrkS-10xERE-NLS4xNG is only detectably expressed after 48h AL2-L3, when ecdysone levels have sufficiently risen. Fluorescence at 0 and 24 hrs originates from the trachea, which cannot be readily dissected away at these stages. **B.** As expected, the control construct (with mutated EREs) is not expressed (Ignore fluorescence in the trachea, which is due to non-specific activity of NLS4xNG reporters). **C-F.** Octβ3R downregulation in the prothoracic gland does not affect 3xGBE-10xERE-NLS levels at the L2-L3 transition (**C-D** n≥5), but strongly reduces reporter activity at mid L3 (**E-F** n≥5). This confirms a previous report [S3] showing that Octβ3R downregulation only affects 20E levels at mid L3. **G-H.** Expression of the 3xGBE-10xERE-NLS4xNG and 3xGBE-10xERE*-NLS4xNG reporters in wing discs at different stages of development. By comparing G and H, we can infer EcR's activity: At 0h, 44h and 48h AL2-L3 it acts as an activator, as the signal is higher in G than H; whereas at 24h AL2-L3 it inhibits the transcription of the reporter. The activity at 0h might be due to the 20E peak that triggers L2 to L3 transition. n≥4 for each of the conditions. Scale bars represent 50 µm.

Supplementary Figure 7

A

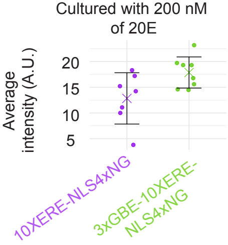

Sup. Fig.7 related to Figure 6. Reporter expression data used for cross experiment comparison.

A. Expression of 10xERE-NLS4xNG and 3xGBE-10xERE-NLS4xNG after 2.5 hr culture in 200nM 20E. At this concentration, 3xGBE-10xERE-NLS4xNG is expressed 1.4 times higher than NLS4xNG. This information was used to allow comparison of the quantifications displayed in Fig. 6 which came from images taken at different times. Error bars represent standard deviation. $n \geq 5$.

ADDITIONAL REFERENCES

S1. Janky, R., Verfaillie, A., Imrichova, H., Van de Sande, B., Standaert, L., Christiaens, V., Hulselmans, G., Herten, K., Naval Sanchez, M., Potier, D., et al. (2014). iRegulon: from a gene list to a gene regulatory network using large motif and track collections. PLoS Comput Biol *10*, e1003731. 10.1371/journal.pcbi.1003731.

S2. Wardwell-Ozgo, J., Terry, D., Schweibenz, C., Tu, M., Solimon, O., Schofeld, D., and Moberg, K. (2022). An EcR probe reveals mechanisms of the ecdysone-mediated switch from repression-to-activation on target genes in the larval wing disc. bioRxiv, 2022.2004.2007.487542. 10.1101/2022.04.07.487542.

S3. Ohhara, Y., Shimada-Niwa, Y., Niwa, R., Kayashima, Y., Hayashi, Y., Akagi, K., Ueda, H., Yamakawa-Kobayashi, K., and Kobayashi, S. (2015). Autocrine regulation of ecdysone synthesis by beta3-octopamine receptor in the prothoracic gland is essential for Drosophila metamorphosis. Proc Natl Acad Sci U S A *112*, 1452-1457. 10.1073/pnas.1414966112.
